# Labile Dissolved Organic Matter Compound Characteristics Select for Divergence in Marine Bacterial Activity and Transcription

**DOI:** 10.3389/fmicb.2020.588778

**Published:** 2020-09-25

**Authors:** Benjamin Pontiller, Sandra Martínez-García, Daniel Lundin, Jarone Pinhassi

**Affiliations:** ^1^Centre for Ecology and Evolution in Microbial Model Systems, Linnaeus University, Kalmar, Sweden; ^2^Departamento de Ecoloxía e Bioloxía Animal, Universidade de Vigo, Vigo, Spain

**Keywords:** labile DOM compounds, carbohydrates, nucleic acids, proteins, bacterial metatranscriptomics, microcosms, brackish water system, functional partitioning

## Abstract

Bacteria play a key role in the planetary carbon cycle partly because they rapidly assimilate labile dissolved organic matter (DOM) in the ocean. However, knowledge of the molecular mechanisms at work when bacterioplankton metabolize distinct components of the DOM pool is still limited. We, therefore, conducted seawater culture enrichment experiments with ecologically relevant DOM, combining both polymer and monomer model compounds for distinct compound classes. This included carbohydrates (polysaccharides vs. monosaccharides), proteins (polypeptides vs. amino acids), and nucleic acids (DNA vs. nucleotides). We noted pronounced changes in bacterial growth, activity, and transcription related to DOM characteristics. Transcriptional responses differed between compound classes, with distinct gene sets (“core genes”) distinguishing carbohydrates, proteins, and nucleic acids. Moreover, we found a strong divergence in functional transcription at the level of particular monomers and polymers (i.e., the condensation state), primarily in the carbohydrates and protein compound classes. These specific responses included a variety of cellular and metabolic processes that were mediated by distinct bacterial taxa, suggesting pronounced functional partitioning of organic matter. Collectively, our findings show that two important facets of DOM, compound class and condensation state, shape bacterial gene expression, and ultimately select for distinct bacterial (functional) groups. This emphasizes the interdependency of marine bacteria and labile carbon compounds for regulating the transformation of DOM in surface waters.

## Introduction

The pool of dissolved organic carbon (DOC) in the ocean represents the largest reservoir of reduced organic carbon in the biosphere (Hansell et al., 2009). This organic carbon is constituted by a myriad of molecules in a continuum of sizes (identified as the range from low- to high-molecular-weight), differing in chemical bonds, element ratios, and condensation states (e.g., being monomers or polymers) (Hansell et al., 2009). These characteristics ultimately determine the timescale of the transformation of DOC components, which can vary over orders of magnitude (i.e., minutes to millennia) (Hansell and Carlson, 2015). Marine bacteria play a key role in regulating Earth’s planetary carbon cycle since they are the key organisms to assimilate and metabolize dissolved organic carbon (DOC) (Azam et al., 1983). The timescales of bacterial transformations are often estimated through combinations of experimental approaches, like short-term incubations measuring bacterial uptake rates of radiolabeled compounds and long-term seawater culture bioassays where the rate and quantity of DOC consumed by bacteria are determined (Allers et al., 2007; Pedler et al., 2014; Lindh et al., 2015b; Osterholz et al., 2015). Collectively, this has resulted in a recognition that, from a perspective of bacterial transformation capacity, parts of the DOC pool in surface waters is in the form of labile DOC–turned over by bacteria within days–whereas parts of the DOC pool is characterized by slower turnover–ranging from weeks or months to decades–often denoted as refractory DOC (Hansell, 2013). The inventory of labile DOC in surface waters is minuscule (<0.2 Gt C) compared to the vast pool of refractory DOC (∼630 Gt C) (Hansell, 2013). Nevertheless, estimates suggest that the transformation of organic carbon to carbon dioxide (CO_2_) is approximately 10 times larger for the labile fraction (∼25 Gt C year^–1^) than for refractory DOC (Hansell, 2013). Consequently, even seemingly small changes in the functional part of the oceanic microbiota are likely to induce significant shifts in the biogeochemical cycling of carbon (Falkowski et al., 1998).

Determining the genetic mechanisms involved in regulating bacterial DOC transformations, and knowledge on how their functional diversity varies in space and time in the ocean remains challenging. Nevertheless, advances in sequencing technology and bioinformatics analysis have initiated an era in which scientists are able to obtain an unprecedented amount of information on the functional processes encoded in the genomes of bacteria (Moran et al., 2016). Accordingly, both field studies and experiments are currently expanding our knowledge on the diversity of bacteria and their functional genes involved in the cycling of carbon (Mou et al., 2008; McCarren et al., 2010; Bergauer et al., 2018; Vorobev et al., 2018; Salazar et al., 2019). Importantly, a majority of these studies indicate a partitioning of DOC resources among a broad range of bacterial key taxa. Genomically encoded traits that distinguish the responses of different bacteria to distinct DOM components include uptake mechanisms (membrane transporters), the acquisition of phosphate and nitrogen, carbohydrates, lipid metabolism, and motility and chemotaxis (McCarren et al., 2010; Poretsky et al., 2010; Beier et al., 2015). Taken together, these studies show the large potential of analyzing the metabolic potential and functional gene expression of bacteria to gain an understanding of the factors that regulate DOC transformations in the ocean.

Even though the precise chemical composition of the marine DOC pool remains a challenging enigma, analyses in microbial oceanography have established that carbon cycling in surface waters is largely dominated by bacterial transformations of labile DOC (Hansell et al., 2009; Moran et al., 2016). This conclusion partly relies on studies of bacterial utilization rates of low molecular weight (LMW) oligomers and organic acids as well as monomeric DOC compounds (e.g., glucose, amino acids, acetate, DMSP, vanillate) that are the building blocks of key biopolymers (Kirchman, 2003; Mou et al., 2008; Alonso-Sáez et al., 2012; Gomez-Consarnau et al., 2012) – to a degree that such compounds have been termed “canonical bacterial substrates” (Poretsky et al., 2010). Further knowledge of bacterial transformations of labile DOC comes from studies on the utilization of polymeric macromolecules like proteins and polysaccharides (McCarren et al., 2010; Beier et al., 2015), but only a few used a combination of both (Bryson et al., 2017). The advantage of the latter is that it allows comparisons between compound classes and between compounds of different condensation states within and between compound classes.

We therefore carried out seawater culture regrowth experiments coupled with metatranscriptomics to identify responses in bacterioplankton activity and functional gene expression toward ecologically relevant DOC monomers and their corresponding polymers–compounds making up major parts of the labile “high-flux” portion of coastal DOM (Biersmith and Benner, 1998; Geider and La Roche, 2002; Kirchman, 2003). The analysis of bacterial responses to these chemically defined DOC compounds with different condensation states enabled us to distinguish: (i) genes that were shared between monomers and polymers of the same compound class (i.e., compound class “core” gene responses), and (ii) genes that were unique to individual monomer or polymer compounds (compound “specific” responses). We infer that such analyses of model DOC compounds have the potential to uncover the molecular mechanisms at work when natural microbial communities meditate the “invisible” flux of carbon associated with labile DOM transformations (Hansell and Carlson, 2015; Moran et al., 2016).

## Materials and Methods

### Study Site, Sampling, and Experimental Setup

Seawater was collected at the Linnaeus Microbial Observatory (LMO) (56°55.840 N, 17°03.640 E), in the Baltic Sea ∼10 km off the northeast coast of Öland, Sweden (Supplementary Figure S1). The water to be used as medium for the seawater culture regrowth experiments was collected with a peristaltic pump (Watson Marlow, Type 620S) from 2 m depth on February 16th, 2016 (experiment 1; water temperature ∼3°C, salinity 7.7 PSU, Chl *a* concentration 0.7 μg L^–1^, phosphate 0.94 μM, nitrate + nitrite 1.1 μM, DOC ∼325 μM) and on March 15th, 2016 (experiment 2; water temperature ∼3.6°C, salinity 7.4 PSU, Chl *a* concentration 3.5 μg L^–1^, phosphate 0.5 μM, nitrate + nitrite ∼1 μM, ammonium ∼0.8 μM, DOC ∼289 μM). These nutrient values are typical for the Baltic Sea in late winter and early spring (Zweifel et al., 1995; Bunse et al., 2019). Note in particular that these DOC concentrations in the brackish Baltic Sea (with high input of river discharge) are generally higher compared to marine coastal waters (with concentrations down to ∼100 μM C) (Hansell et al., 2009; Barrón and Duarte, 2015). The water was collected in a 64 L HDPE drum (Curtec International, Netherlands) and 10 L polycarbonate carboys (Nalgene, Thermo Fisher Scientific) until further processing in the laboratory at the Linnaeus University (LNU), Kalmar, Sweden. The medium collected on March 15th was stored in a temperature-controlled room at ∼8.5°C in the dark until the start of experiment 2 on March 31st.

**FIGURE 1:**
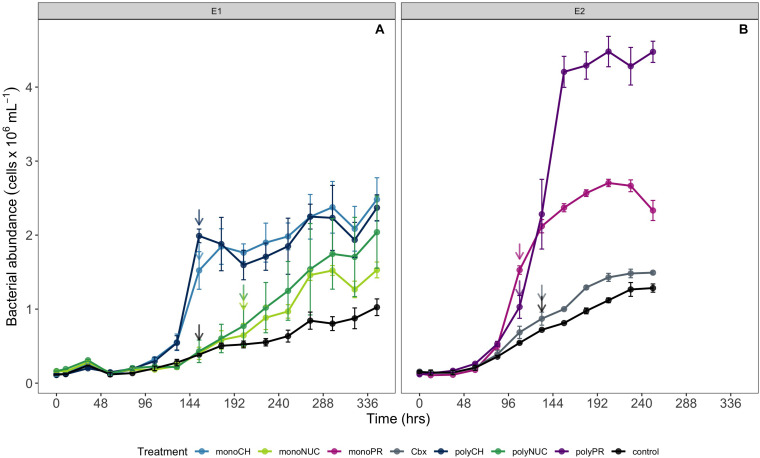
Microbial community growth dynamics during incubations determined by flow cytometry **(A)** experiment 1 and **(B)** experiment 2. Cell counts are shown as averages of biological triplicates ± standard deviation (SD) × 10^6^ obtained in experiment 1 **(A)** and 2 **(B)**. Arrows indicate the time points when samples for metatranscriptomics were taken. Samples for metatranscriptomic analysis were retrieved after 108 h (monoPR, amino acids; and polyPR, polypeptides), 132 h (Cbx, carboxylic acids; and contTwo, control from experiment 2), 154 h (monoCH, monosaccharides; polyCH, polysaccharides; and contOne, control from experiment 1), and 202 h (monoNUC, nucleotides; and polyNUC, DNA).

Seawater samples for bacterial inocula for the seawater cultures were retrieved from 2 m depth using a 5 L Ruttner sampling device on February 16th and March 31st, 2016 for experiment 1 and experiment 2 at ∼9:30 a.m., respectively. We performed each of the experiments on the same day that we collected bacterial inocula. Experiment 1 started on February 16th with seawater medium collected on the same date, whereas experiment 2 was started on March 31st with seawater medium collected in the field from March 15th—this was due to logistical reasons with obtaining larger amounts of water from the LMO station. Vertical profiles (i.e., salinity, temperature, and fluorescence) of the water column, down to ∼40 m depth, were taken during all *in situ* samplings with a CTD probe (AAQ 1186-H, Alec Electronics, Japan). Seawater culture enrichment experiments were conducted in acid rinsed (1 M HCl) 10 L polycarbonate carboys (Nalgene) and incubated in a temperature-controlled room at 8.5°C. In brief, medium was prepared by filtration of *in situ* seawater through sterivex filter units (GP 0.22 μm, EMD Millipore). For the medium, 8.45 L of “bacteria-free” water was spiked with 0.45 L of 0.6 μm (Whatmann, polycarbonate filters, 47 mm diameter) filtered seawater (experiment 1: 5.3 × 10^5^ cells mL^–1^ and experiment 2: ∼1 × 10^6^ cells mL^–1^) (Supplementary Table S1), to obtain an initial cell abundance of ∼5 × 10^4^ cells mL^–1^ in the seawater culture regrowth experiments (Figures 1A,B). In order to ensure that bacterial growth was not limited by inorganic nutrients (N or P), biological triplicates were spiked with 5 μM ammonium chloride (NH_4_Cl) and 0.5 μM sodium phosphate (NaH_2_PO_4_) in a ratio of 10:1, N and P, respectively according to (Zweifel et al., 1993).

In experiment 1, seawater culture enrichments with organic compounds were composed of a monosaccharide, polysaccharide, nucleotide, and a DNA treatment. The monosaccharide (monoCH) treatment was a mixture of D-(+)-Glucose (G5767), D-(+)-Galactose (G5388), D-(+)-Mannose (M6020), L-Rhamnose (W373011), D-(+)-Xylose (W360600) and D-(-)-Arabinose (A3131), while the polysaccharide (polyCH) treatment was composed of Glycogen Type II from Oyster (G8751) and Starch (33615) (Table 1). The nucleotide treatment (monoNUC) consisted of a nucleotide triphosphate (dNTP) mix (D7295) and the DNA treatment (polyNUC) consisted of DNA from fish sperm (74782) (Table 1). In experiment 2, we tested a polypeptide treatment (polyPR) that received Bovine Serum Albumin (BSA; A2153), a treatment with a mixture of amino acids (monoPR) consisting of 20 amino acids (09416-1E) that were added in the same ratios as present in BSA, and a treatment with a mixture of carboxylic acids (Cbx) consisting of Formate (71539), Acetate (S2889), Propionate (P5436), Pyruvate (P5280), Glyoxylic acid (G10601), and Glycolic acid (124737) (Table 1). All organic compounds used in this study were purchased from Sigma-Aldrich. Microcosms (acid-rinsed 10 L polycarbonate carboys) were spiked with the respective compounds in non-limiting concentrations (final concentration added per replicate was ∼10 μM C). Actual measurements of DOC concentrations in the bottles confirmed this level of enrichment, with DOC concentrations around ∼320 μM C for most treatments as compared to ∼310 μM C in controls, but with no increase in the carboxylic acids treatment (303 μM C) (Supplementary Table S2). This level of DOC enrichment corresponds to reported values on the labile portion of DOC present in the Baltic Sea and other coastal waters (Zweifel et al., 1993; Sondergaard and Middelboe, 1995). The controls for both experiment 1 and experiment 2 consisted of 0.22 μm filtered water (“bacteria-free”) and received the same concentrations and ratio of inorganic nutrients (ammonium chloride and sodium phosphate) as the treatments. All treatments and controls were tested in biological triplicates (*n* = 3).

**TABLE 1:** Overview of the investigated organic matter compounds.

**Compound class**	**Condensation state**	
	**Monomers**	**Polymers**
Carbohydrates (CH)	Glucose, galactose, mannose, rhamnose, xylose, arabinose	Glycogen, starch
Nucleic acids (NUC)	Deoxyadenosine triphosphate (dATP), Deoxycytidine triphosphate (dCTP), Deoxyguanosine triphosphate (dGTP), Thymidine triphosphate (dTTP)	DNA (fish sperm)
Protein (PR)	L-Alanine, L-Arginine hydrochloride, L-Asparagine, L-Aspartic acid, L-Cysteine, L-Cystine, L-Glutamic acid, L-Glutamine, Glycine, L-Histidine hydrochloride, L-4-Hydroxyproline, L-Isoleucine, L-Leucine, L-Lysine hydrochloride, L-Methionine, L-Phenylalanine, L-Proline, L-Serine, L-Threonine, L-Tryptophan, L-Tyrosine, L-Valine	Bovine serum albumin (BSA)
Carboxylic acids (Cbx)	Formate, acetate, propionate, pyruvate, glyoxylic acid, glycolic acid	NA

**NA, Not available (tested). Further details are shown in section “Materials and Methods”.**

### Microbial Cell Abundance

Cell counts for bacterial inocula were performed immediately after water was collected and filtered (February 16th for experiment 1 and March 31st for experiment 2) (Supplementary Table S1). Subsequently, enrichment experiments were conducted, and we started experiment 1 on February 16th and it lasted for 346 h (∼15 days). Experiment 2 was started on March 31st and it lasted for 252 h (∼11 days). Samples (biological triplicates) for bacterial cell counts were preserved daily and analyzed by flow cytometry (CyFlow Cube 8, Sysmex Partec). Briefly, 1.8 mL of sample was fixed with 1% paraformaldehyde and 0.05% glutaraldehyde (final), stained with SYBR Green I nucleic acid stain (Invitrogen) (5 μM final concentration), and spiked with 2 μL Flow Check High Intensity Green Alignment beads (Polysciences Inc.) with a diameter of 1 μm as an internal standard. Subsequently samples were incubated for 15 min in the dark at room temperature (RT) prior to counting. Bacterial cell counts were plotted in cytograms, green fluorescence (FL1) vs. side scatter (SSC) and particle counts converted to cells mL^–1^ as described in delGiorgio et al. (1996) and discussed in Gasol and Del Giorgio (2000).

### Nutrient Analysis

*In situ* nutrient concentrations (i.e., NO_3_ + NO_2_, NH_4_, PO_4_) (water used for seawater medium) were determined using colorimetric methods (UV-1600 Spectrometer, VWR) as described in Valderrama (1995). Chlorophyll *a* (Chl *a*) concentrations in the field were determined fluorometrically upon ethanol extraction according to Jespersen and Christoffersen (1987) as described in Bunse et al. (2019). Samples to determine dissolved organic carbon (DOC) concentrations were taken at the LMO station during the time of sampling for medium. Additionally, samples were taken from each microcosm replicate immediately after the addition of organic compounds (T0). DOC concentrations were determined as follows, 30 mL of sample was filtered through 0.2 μm syringe filters (Acrodisk syringe filters, 32 mm, 514–4131, VWR), filled in MQ washed 60 mL EasY flasks (Nunc^TM^ Cell Culture Treated EasYFlasks^TM^ 25 cm^2^, 156340, Thermo Fisher Scientific) and acidified with 448 μL of 1.2 M hydrochloric acid to a pH of ∼2. Flasks were stored in darkness at 4°C until analysis with a high-temperature carbon analyzer (Shimadzu TOC-5000) at the Umeå Marine Science Centre, Umeå, Sweden. Finally, DOC concentrations (one replicate per replicate enrichment culture bottle) were calculated as non-purgeable organic carbon and mean values per treatment calculated (*n* = 3).

### Total Extracellular Enzymatic Activity

The total microbial extracellular enzymatic activity (EEA) during the enrichment regrowth experiments was determined in regular intervals (in experiment 1 after 34, 106, 178, 250, and 322 h and in experiment 2 after 36, 108, 180, 252, and 300 h). The hydrolysis rates of fluorogenic substrate analogs 4-methylumbelliferyl (MUF) and 4-methylcoumarinyl-7-amide (MCA) were used to estimate the hydrolysis rate of (MUF)-β-D-glucosidase (BGase), (MUF)-α-D-glucosidase (AGase), (MUF)-alkaline phosphatase (APase) and (MCA)-L-leucine aminopeptidase (LAPase) according to Hoppe (1983) as described in Baltar et al. (2016) and references therein. In brief, all EEA assays were conducted in 96-well plates and measured at 365 nm excitation (EX) and 445 nm emission (EM) wavelength with a microwell plate reader (FLUOStar, BMG Labtech). Substrate analogs were measured in three technical replicates per culture bottle in experiment 1 and in five technical replicates per culture bottle in experiment 2 (300 μL final volume per well) within 30 min after subsamples were taken (T0), incubated at ∼8.5°C temperature in the dark for ∼2 h, and measured again (Tf). Subsamples for blanks were filtered through 0.2 μm low protein binding filters (Acrodisc, Pall) and fluorescence values blank corrected. The hydrolysis rates of total extracellular enzymatic activities were calculated by using standard curves that were obtained by using various concentrations of the fluorogenic substrates MUF and MCA. The substrates were diluted with blank water and run separately on each 96-well plate. The hydrolysis rates were calculated based on the fluorescence increase over the incubation time (Tf—T0). Finally, hydrolysis rates were averaged over the technical replicates and mean values per treatment (*n* = 3) calculated. The final substrate concentrations (previously determined as saturating concentrations (Baltar et al., 2016) used were 32.25 μM for MUF-AGase and MUF-BGase, 100 μM for MUF-APase and 500 μM for MCA-LAPase.

### Bacterial Production

Subsamples for bacterial production in experiment 1 were taken after 34, 106, 178, 250, and 322 h and in experiment 2 after 36, 108, 180, 252, and 300 h. Bacterial production was estimated through tritiated leucine incorporation according to (Smith and Farooq, 1992). A 1:10 hot:total leucine solution was prepared (final concentration 1 μM leucine) and triplicate samples (1.2 mL) were amended with 48 μL of the radioactive leucine solution (final concentration of 40 nM leucine) (specific radioactivity ∼153 Ci mmol^–1^, Perkin Elmer) in 2 mL microtubes (Sarstedt). Samples were incubated at *in situ* temperature in the dark for 2 h, and subsequently leucine incorporation terminated by spiking the samples with 50% trichloric acid (TCA) to a final concentration of 5%. In addition, one blank (killed control) per sample was prepared and after leucine addition designated samples immediately killed with 50% TCA (final concentration 5%). All samples were stored at −20°C until further processing. Samples were thawed at room temperature (RT) and centrifuged for 10 min at 12,000 g, supernatants were aspirated and the cell pellets washed two times in 5% TCA. Subsequently, the cell pellets were resuspended in 1 mL liquid scintillation cocktail (FilterCount, Perkin Elmer) and incubated for ∼24 h at RT in the dark. The radioactivity incorporated into cells was determined in a liquid scintillation counter (Tri-Carb 2100TR, Packard). The average disintegration per minute (DPM) of technical triplicates were blank corrected. Finally, the bacterial production was calculated with a cellular carbon to protein conversion factor of 0.86 kgC mol leu^–1^, an assumed proportion of leucine in total protein of 0.073%, and an isotopic dilution factor of 2 according to (Simon and Azam, 1989). The mean of technical triplicates was averaged for each treatment (*n* = 3).

### RNA Collection and Extraction

**FIGURE 2:**
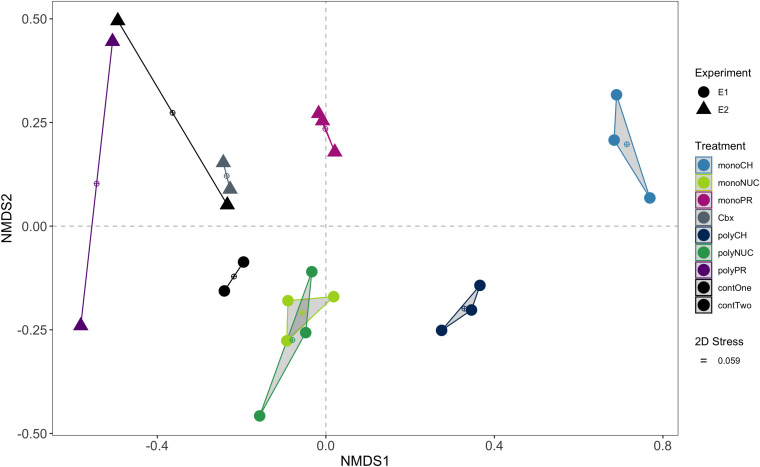
Non-metric multidimensional scaling (NMDS) ordination of functional genes based on pairwise Bray-Curtis distances of normalized counts per million (cpm). Convex hulls group biological replicates within treatments. Experiment 1, E1 (conducted on February 16th, 2016); experiment 2, E2 (performed on March 15th, 2016); monoCH, monosaccharide mix; monoNUC, nucleotide mix; monoPR, amino acid mix; Cbx, carboxylic acid mix, polyCH, polysaccharide mix; polyNUC, DNA; polyPR, polypeptides; contOne and contTwo, controls from experiment 1 and 2, respectively.

Water for subsequent metatranscriptomic analysis was retrieved after 154 h (hours) during experiment 1 from microcosms with monosaccharides (monoCH; *n* = 3), polysaccharides (polyCH; *n* = 3) and from the control from experiment 1 (contOne; *n* = 2). Nucleotide (monoNUC; *n* = 3) and DNA (polyNUC; *n* = 3) were filtered after 202 h of incubation (Figures 1A, 2). The bacterial development in experiment 2 was slightly faster, samples were taken after 108 h from amino acids (monoPR; *n* = 3) and polypeptides (polyPR; *n* = 2) and after 132 h from carboxylic acids (Cbx; *n* = 2) and from the second control (contTwo; *n* = 2) (Figures 1B, 2). Approximately 3.5 L of water was filtered through Sterivex filter units (GP 0.22 μm, EMD Millipore), preserved in 2 mL RNAlater (Qiagen), immediately flash frozen in liquid nitrogen and stored at -80°C until further processing. Total RNA was extracted according to (Poretsky et al., 2009) with the RNeasy Mini Kit (Qiagen) and minor adaptations. In brief, Sterivex filter units were thawed on ice at room temperature for 30 min. Subsequently, RNAlater was removed by using a sterile 20 mL syringe and applying gentle pressure until all RNAlater was removed from the sterivex filter cartridges. The inner part of the sterivex filter that holds the filter membrane was cut loose using a sterile razor cutter. Filters were gently transferred into 15 mL Falcon tubes containing a solution of 4 mL RLT lysis buffer +β-Mercaptoethanol (10 μL/mL RLT buffer) and 1.5 g of 200 μm zirconium beads (OPS diagnostics). Cells were disrupted by vortexing the tubes for 15 min at room temperature (Genie II, Scientific Industries), followed by centrifugation for 5 min at 3,260 g, supernatants were transferred to tubes containing 1 volume of 70% ethanol, and each sample was thoroughly mixed by pipetting several times. RNA extraction and purification were performed with RNeasy Mini Kits according to manufacturer’s instructions and each sample was divided into two spin columns. Total RNA was eluted two times with 35 μL of preheated (50°C) RNase-free water. Each sample was DNase treated using TURBO DNA-free Kit to remove residual DNA (Thermo Fisher Scientific) according to the manufacturer’s protocol. Samples were tested for remaining DNA by a 30-cycle PCR with 16S rDNA primers (27F and 1492R) and a 1% agarose gel with bleach (final con. 0.12%) according to (Aranda et al., 2012) and the gel stained with ethidium-bromide. RNA yield was measured using NanoDrop 2000 (Thermo Fisher Scientific), and fluorometric quantification of RNA concentration was assessed with Quibit 2.0 (Invitrogen). Prior to RNA amplification, the best technical replicate from each sample was selected for further processing based on NanoDrop and Qubit values. Ribosomal RNA was depleted using RiboMinus Transcriptome Isolation Kit and RiboMinus Concentration Module (Thermo Fisher Scientific) according to the manufacturer’s protocols. Shortly, ribosomal RNA molecules were removed by binding to streptavidin-coated magnetic beads and only the rRNA-depleted fraction was further concentrated and purified using silica-based spin columns. RNA was linearly amplified using the MessageAmp II-Bacteria RNA Amplification Kit (Thermo Fisher Scientific) according to manufacturer’s instructions and finally converted into cDNA and quantified using NanoDrop and Qubit, and stored at −80°C until TruSeq library construction and sequencing at the National Genome Infrastructure, SciLifeLab Stockholm on an Illumina HiSeq 2500 platform in rapid mode and with v3 chemistry to obtain 2 × 125 bp long paired-end reads.

### Metatranscriptome Data Pre-processing and Annotation

The paired-end reads (2 × 125 bp) were quality checked with FastQC and MultiQC. Illumina adapter sequences were removed with Cutadapt (Martin, 2011) version 1.13 with a maximum error rate threshold of 0.1 (10%). Thereafter, reads were trimmed with Sickle (Joshi and Fass, 2011) version 1.33 in paired-end mode and with sanger quality values. Afterward, ribosomal RNA was bioinformatically filtered with ERNE (Del Fabbro et al., 2013) version 2.1.1 against an in-house database of stable RNA sequences from marine microbes. Subsequently, forward and reverse reads were merged with PEAR (Zhang et al., 2014) version 0.9.10 with a minimum assembly length of 50 nt, *p*-value 0.01, and a minimum overlap of 10 nt. Merged reads were subsequently aligned with DIAMOND (Buchfink et al., 2015) version 0.8.26 against the NCBI RefSeq protein database with a default *e*-value cutoff of 0.001. Subsequently, functional SEED classification and taxonomic affiliation were assigned with MEGAN (Huson et al., 2016) version 6.7.3. Summary statistics can be found in Supplementary Table S3. Finally, differential gene expression (DGE) analysis was conducted using the package *edgeR* (Robinson et al., 2010) according to (Chen et al., 2016).

### Multivariate Analysis (NMDS and PERMANOVA)

Relative raw gene counts were normalized to library sizes and denoted in counts per million (cpm). Pairwise Bray-Curtis distances were calculated between samples and NMDS ordination computed with the *metaMDS* function in *vegan* (Oksanen et al., 2019). The influence of compound class and condensation state on the bacterial community gene expression was tested using a permutational multivariate analysis of variance (PERMANOVA) with the function *adonis* from the vegan package. Also, we tested for multivariate homogeneity of group dispersions (a multivariate analog of Levene’s test for homogeneity of variances) with the functions *betadisper*, *permutest*, and *TukeyHSD* from the *vegan* package. These tests allow teasing apart whether groups differ because of differences in their centroids or dispersion. Given that these homogeneity tests were non-significant for compound class (*p* > 0.17) and condensation state (*p* > 0.92) based on 999 permutations, the distinction between the groups as tested by PERMANOVA can safely be attributed to differences in centroids.

### Differential Gene Expression Analysis (EdgeR)

Differential gene expression analysis was performed according to Chen et al. (2016) with the R package *edgeR*. In brief, genes with five or more cpm counts in at least two samples were normalized for compositional bias by trimmed mean of *M*-values (TMM). Libraries were corrected for differences in library size (norm.lib.sizes = TRUE). Thereafter, a design matrix was created to specify pairwise statistical comparisons between each of the treatments and controls. A global dispersion averaged over all genes was estimated with the function *estimateDisp* and we accounted for possible outlier genes with exceptionally large or small individual dispersions (robust = TRUE). Moreover, to account for gene-specific variability from both biological and technical sources, the negative binomial (NB) model was extended with quasi-likelihood (QL) methods by using generalized linear models and empirical Bayes quasi-likelihood F-tests with the functions *glmQLFit* and *glmQLFTest*, respectively, as described in Chen et al. (2016). Finally, genes were considered to be significantly differentially expressed if their log2-fold change was positive in treatments and their associated false discovery rate (FDR) (*p*-values corrected for multiple testing according to Benjamini Hochberg) below 5%.

### Definitions and Thresholds

For practical reasons we shortened the following SEED category names *Cofactors, Vitamins, Prosthetic Groups, Pigments*; *Virulence, Disease and Defense*; *Fatty Acids, Lipids, and Isoprenoids*; *Amino Acids and Derivatives*. Instead, we refer to them as *Vitamins*; *Virulence*, *Fatty Acids and Lipids*, and *Amino Acids* in the text. We also decided to include the SEED category *Thiamin* in *Vitamins* and *Polyamines* in *Amino Acids*. In addition to the original SEED classification hierarchy, we added entries which matched “transport” and “permease” at the lowest seed level “gene” name to the category *Membrane Transport* to account for biases originating from the single assignment of for example carbohydrate-specific transporters which were only present in the *Carbohydrate* category.

### Selection of Genes Assigned to Core and Non-core Metatranscriptomes

For this analysis, we first verified the consistency of the control replicates by using an outlier test with the function *voomWithQualityWeights* from the *limma* R package. This test showed that controls in experiment 1 were appropriate (threshold > 1) but identified the control replicate E2_C1 in experiment 2 as an outlier (threshold = 0.29). Next, for experiment 2, given that gene expression in the carboxylic acids (Cbx) treatment appeared to be similar to the controls (as seen in the NMDS Figure 2), we statistically tested the similarity of these groups using a differential gene expression analysis (DGE in edgeR) between carboxylic acids (Cbx; *n* = 2) relative to controls (from experiment 2; *n* = 2). This test showed no significant difference in expressed genes between Cbx and the controls at a 5% false discovery rate (FDR) threshold (data not shown). This was in line with the results on the similarity between Cbx and controls in experiment 2 in bacterial growth and DOC concentrations (see Figure 1 and Supplementary Table S2). We, therefore, combined the carboxylic acids treatment with the non-outlier control from experiment 2 to increase the statistical power of our analysis of the protein treatments (i.e., monoPR, *n* = 3 and polyPR, *n* = 2 vs. the composite control, *n* = 3 consisting of the two Cbx samples and one control replicate).

**FIGURE 4:**
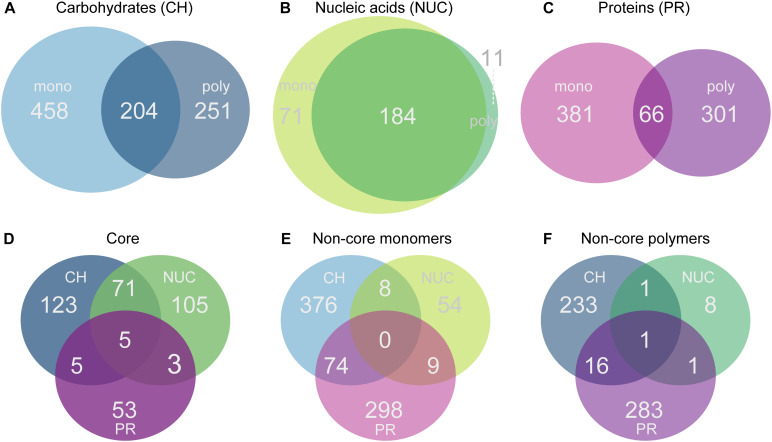
Venn diagrams showing the number of genes that were significantly overrepresented at a 5% false discovery rate (FDR) threshold relative to controls. Comparisons were made for each treatment within a compound class against the controls. Genes were selected according to the significance and the same change in direction (positive log_2_-fold-change). **(A)** Monosaccharide additions compared with polysaccharides, **(B)** nucleic acids vs. DNA; **(C)** amino acids vs. polypeptides, **(D)** shows the comparison of the three different compound class core sets of genes (carbohydrates, nucleic acids, protein), **(E)** shows the comparison of all monomer non-core genes, and **(F)** the polymer non-core comparisons across all compound classes with a positive change in direction.

Differential gene enrichment analysis was performed with *edgeR* and statistical pairwise comparisons made between each treatment and the controls: monoCH (*n* = 3) vs. contOne (*n* = 2); polyCH (*n* = 3) vs. contOne (*n* = 2); monoNUC (*n* = 3) vs. contOne (*n* = 2); polyNUC (*n* = 3) vs. contOne (*n* = 2); monoPR (*n* = 3) vs. composite control (*n* = 3), and polyPR (*n* = 2) vs. composite control (*n* = 3). To identify core and non-core transcriptomes, (i) we considered genes that had a positive log2-fold-change in these pairwise statistical tests (see above), as responsive to organic matter enrichments. (ii) Thereafter, we compared these sets of responsive genes between condensation states (i.e., monomers vs. polymers for each compound class separately) to define core sets of genes (i.e., the compound class responses) consisting of genes that were present in both monomer and polymer treatments with a false discovery rate (FDR) below 5% (overlaps in Figures 4A–C). (iii) Genes that had an FDR below 5% in either monomers or polymers (but not in both) were assigned to the non-core (i.e., monomer- or polymer-specific non-core responses) (Figures 4A–C). (iv) Also, we compared the three different core sets of genes between compound classes (overlaps of Figures 4A–C shown in Figure 4D) and similarly the respective monomer- and polymer-specific non-cores separately (Figures 4E,F).

### Functional Signature Analysis

To obtain a broad overview of important functions that are involved in the processing (i.e., degradation and modification) and uptake of the added canonical carbon compounds – here referred to as “functional signatures” – we grouped significantly differentially abundant genes (<5% FDR) according to their enzyme classes (EC numbers) present in the carbohydrate-active enzyme (CAZyme) database (last updated on June 26th, 2020) into CAZyme classes (glycoside hydrolases, glycosyltransferases, carbohydrate esterases, polysaccharide lyases, auxiliary activities, and carbohydrate-binding modules), peptidases (EC 3.4), transferases (2.1–2.10) and transporter associated genes based on their transporter classification numbers (TC) when available, in addition to matching seed names containing the search string “transporter” or “permease,” regardless of the assigned SEED hierarchy.

### Statistics and Graphics

All statistical and graphical analyses were performed using RStudio Version 1.2.5019 (RStudio Team, 2019) and analyses primarily relied on the packages *vegan*, *edgeR*, *ggplot2*, and other packages included in the *tidyverse* (Wickham, 2017).

## Results

### Bacterial Growth Dynamics

We studied bacterial responses to enrichments with ecologically relevant carbon compounds, belonging to different compound classes and represented by monomer and polymer forms (see list of compounds in Table 1). The studied compounds were selected to represent DOM components that are generally recognized to be important substrates to marine bacteria (Kirchman, 2003; Moran et al., 2016; Muhlenbruch et al., 2018). Moreover, we have previously shown that bacteria in our study site in the Baltic Sea actively take up several of the monomers (i.e., glucose, mixed amino acids, leucine, pyruvate, and acetate) that we used here (Bunse et al., 2019).

The current results showed that the compounds studied here consistently stimulated bacterial growth compared to controls. Growth in the carbohydrates treatments was similar with cell yields reaching ∼2.5 ± 0.3 × 10^6^ cells mL^–1^ (mean ± SD) (Figure 1A). Growth in the two nucleic acids (NUC) treatments was slower compared to carbohydrates, with yields up to 2.0 ± 0.5 × 10^6^ cells mL^–1^ (Figure 1A). The highest cell yield was obtained in the polypeptide treatment at ∼4.5 ± 0.2 × 10^6^ cells mL^–1^, nearly double the cell abundance in the amino acid treatment (Figure 1B). Growth yields with carboxylic acids were only marginally higher (∼1.5 × 10^6^ cells mL^–1^) than in the controls (Figure 1B).

### Extracellular Enzymatic Activities

α-glucosidase (AGase) and β-glucosidase (BGase) activities in the polysaccharide (polyCH) treatment were 8- and 2-fold higher, respectively, compared to the monosaccharide (monoCH) treatment (AGase in polyCH peaked at 80 ± 39.7 nM h^–1^ and BGase 18.4 ± 5.3 nM h^–1^, mean ± SD), and were substantially lower in the other treatments (below 7 nM h^–1^) (Supplementary Figures S2A,B). Leucine aminopeptidase (LAPase) activity was highest in amendments with polypeptides (polyPR) (∼4,000 ± 147 nM h^–1^) and ∼7-fold lower in amino acids, and below 500 nM h^–1^ in the other treatments (Supplementary Figure S2C). Moreover, in carbohydrates and nucleic acids treatments, alkaline phosphatase (APase) hydrolysis was below 200 nM h^–1^, but relatively constant in protein and carboxylic acids amendments (Supplementary Figure S2D).

The AGase activity normalized per cell was highest in polysaccharides (∼42 fM cell^–1^ h^–1^), ∼10-fold lower in monosaccharides, and never exceeded 24 fM cell^–1^ h^–1^ in the other treatments (Supplementary Figure S2E). The per-cell BGase hydrolysis was highest in monosaccharide (∼43 fM cell^–1^ h^–1^) without a clear pattern associated with a certain compound class or condensation state (Supplementary Figure S2F). Cell-specific LAPase activity was high in the polypeptide treatment (∼900 fM cell^–1^ h^–1^) and generally lower in amino acids although it increased ∼10-fold toward the end of the experiment (Supplementary Figure S2G). Interestingly, cell-specific APase hydrolysis was highest in amino acid treatments at the final time points reaching up to ∼1500 fM cell^–1^ h^–1^, but only ∼40 fM cell^–1^ h^–1^ in polypeptides. APase hydrolysis in the other treatments, including polypeptides, was highest at the start of incubations (∼800 fM cell^–1^ h^–1^) (Supplementary Figure S2H).

### Bacterial Production

Amendments with labile organic carbon compounds stimulated bacterial production (BP) in all treatments (Supplementary Figures S3A,B). However, BP differed considerably in the protein treatments, peaking at ∼140 μgC L^–1^ d^–1^ in amino acid but ∼4-fold lower in polypeptide treatments (Supplementary Figure S3B). The bacterial production in the carbohydrate treatments reached a maximum at ∼85 μgC L^–1^ d^–1^ in the monosaccharides (Supplementary Figure S3A). Production in the nucleic acid treatments was even lower but still higher than in the controls (Supplementary Figure S3A).

The cell-specific bacterial production peaked at ∼100 fgC cell^–1^ d^–1^ in carbohydrates and in the amino acid treatments (Supplementary Figures S2C,D) followed by DNA (∼65 fgC cell^–1^ d^–1^). In experiment 1, bacterial production increased toward the end in the controls, reaching ∼66 fgC cell^–1^ d^–1^ (Supplementary Figure S3C).

### Divergence in Functional Gene Expression Upon Substrate Additions

An NMDS analysis of bacterioplankton community transcription based on 3,389 genes showed that biological replicates mostly grouped together (Figure 2). The largest differences from controls, and also between monomers and polymers, were found for expression in the carbohydrate treatments. Considerable differences were also observed between monomers and polymers for the protein treatments. In contrast, nucleic acid amendments clustered away from the controls and monomers and polymers of this compound class overlapped in the NMDS (Figure 2). The carboxylic acid samples were grouped together with one replicate of the controls (Figure 2).

To statistically investigate the groupings observed in the NMDS analysis, we tested the influence of compound class (i.e., carbohydrates, nucleic acids, and proteins) and the effect of condensation state (i.e., monomers or polymers) using permutational multivariate analysis of variance (PERMANOVA) with the three major compound classes where both forms (monomers and polymers) were available. This analysis showed that compound class significantly explained major portions of the variation in gene expression (*df* = 2, *R*^2^ = 0.48, *p* = 0.0001) and an additional portion was significantly explained by the condensation state (*df* = 1, *R*^2^ = 0.11, *p* = 0.0001). However, there was a significant interaction between the terms “compound class” and “condensation state” (*df* = 2, *R*^2^ = 0.22, *p* = 0.0001), showing that the variation attributed to condensation state strongly depended on compound class. Thus, the low *R*^2^ value for condensation state was likely caused by the similarity in expression between the nucleic acids monomer and polymer treatments.

### Taxon-Specific Gene Expression Responses Inferred From Functional Gene Read Abundance

**FIGURE 3:**
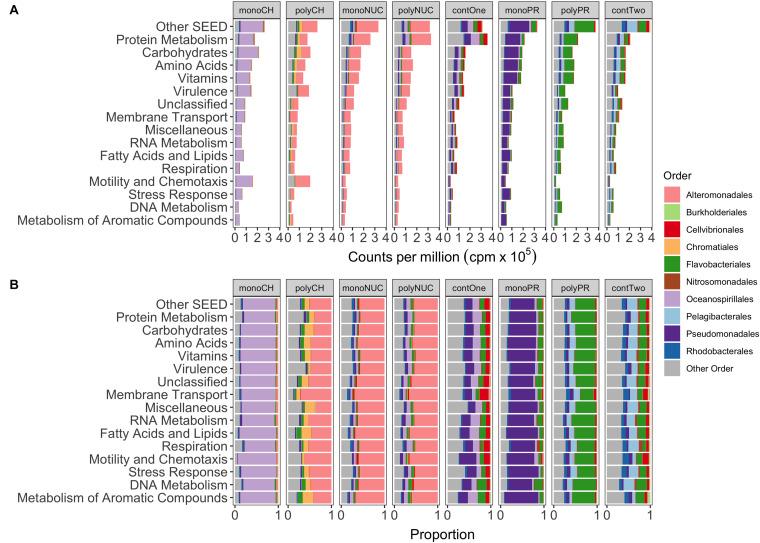
Functional gene expression of the 10 most abundant orders grouped into the 15 most abundant metabolic SEED categories. **(A)** Shows the mean normalized counts per million (×10^5^) of biological replicates. **(B)** Depicting proportions of normalized read counts. monoCH, monosaccharide mix (*n* = 3); polyCH, polysaccharide mix (*n* = 3); monoNUC, nucleotide mix (*n* = 3); polyNUC, DNA (*n* = 3); contOne, untreated seawater from experiment 1 (*n* = 2); monoPR, amino acid mix (*n* = 3); polyPR, polypeptides (*n* = 2); and contTwo, composite control from experiment 2 (*n* = 4, see also section “Materials and Methods”).

Taxonomic analysis of reads grouped into top-level SEED categories showed distinct responses in bacterial gene expression between compound classes and condensation states (mono- and polymers) of the same compound class, with the exception of the nucleic acids treatments (Figures 3A,B). In the monosaccharide treatment, Oceanospirillales largely dominated the expression, whereas Alteromonadales dominated in the polysaccharide treatment together with some smaller portions of Chromatiales and Flavobacteriales (Figure 3A). Alteromonadales were highly dominant in the two nucleic acids treatments. In the amino acid treatment, Pseudomonadales dominated together with some minor portion of Flavobacteriales. Instead, the Flavobacteriales dominated expression in polypeptides with Pelagibacterales and other taxa (Figure 3A). Notable was that expression in the controls was more varied compared to the treatments, where controls from experiment 1 (contOne) had a larger contribution of Pseudomonadales and Oceanospirillales but also Cellvibrionales, and controls from experiment 2 (contTwo) of Pelagibacterales and Rhodobacterales and other taxa (Figures 3A,B).

Oceanospirillales expression was conspicuous in particular in the categories *Carbohydrates* (17.5% of total reads), *Motility and Chemotaxis*, and *Protein Metabolism* (∼13% each), whereas, in polysaccharides, a majority of genes was expressed by Alteromonadales in the *Motility and Chemotaxis* (∼12%), *Virulence*, and *Carbohydrates* (∼8% each) (Figure 3B) categories. In addition, Chromatiales were active in the *Carbohydrates* (∼4%), *Amino Acids*, and *Protein Metabolism* (∼2% each). The nucleic acid treatments with Alteromonadales dominance were highly similar to each other (both in relative gene expression in SEED categories and taxonomic gene expression profiles). The superiority of Alteromonadales not only in polysaccharides but also in the nucleic acid treatments was interesting, contributing in the *Protein Metabolism* (∼15%), the *Amino Acids* and *Carbohydrates* (∼10% each), but surprisingly, only minor in *Motility and Chemotaxis* (∼2%) (Figure 3B). Expression of Pseudomonadales affiliated genes in the amino acid treatment peaked in *Protein Metabolism* (13%), *Carbohydrates* and *Amino Acids* (∼12%), whereas Flavobacteriales in polypeptide treatments had higher expression in *Protein Metabolism* (∼16%), *Vitamins*, *Amino Acids*, *Carbohydrates* (∼10% each), with an interesting shift in *Membrane Transport* (∼2%) (Figure 3B).

### Distribution of Gene Expression Responses Into Core and Non-core Sets of Genes

Next, we aimed at identifying potential gene expression patterns shared between the corresponding polymer and monomer treatments of carbohydrates, nucleic acids, and proteins, i.e., core gene expression responses within the studied compound classes. We thus made pairwise statistical comparisons of gene expression levels between the treatments and the controls using *edgeR* and compared the lists of significant genes between monomer and polymer treatments. Interestingly, this analysis allowed distinguishing both genes that were part of a shared response to different compound classes (i.e., core genes) and genes that were specific to each monomer and polymer treatment within the respective compound classes.

**FIGURE 5:**
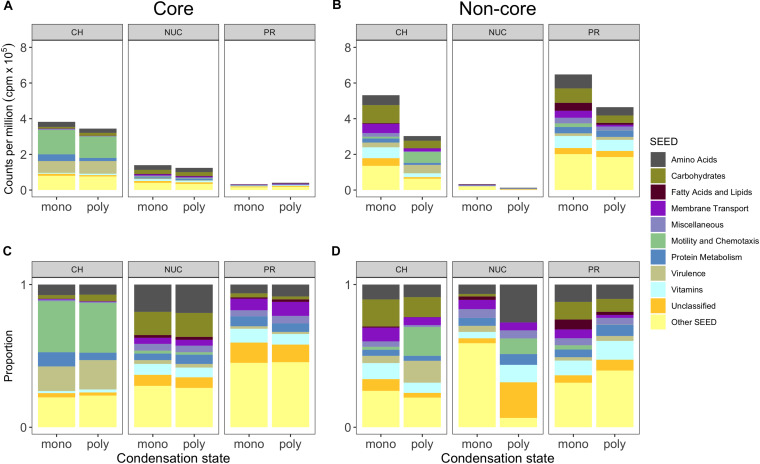
Core and non-core gene expression responses grouped into metabolic SEED categories depicting compound classes and condensation states. **(A)** Shows the mean (*n* = 3, except polyPR *n* = 2) of normalized counts per million (cpm) × 10^5^ of biological replicates grouped into the 10 most abundant metabolic SEED categories of the compound class “core” and **(B)** the “non-core”. **(C,D)** Show the proportions shown in the panels above for improved visibility of the SEED categories. Genes were selected based on statistical pairwise comparisons of each treatment within a compound class against the controls. A significant overrepresentation of genes in treatments is based on a 5% false discovery rate (FDR) and a significantly positive change in relative abundance (direction). Abbreviation of compound classes: CH, carbohydrates; NUC, nucleic acid; PR, proteins. Abbreviation of condensation state: mono, monomers; poly, polymers.

In each of the compound classes investigated, the levels of expression (summed relative abundances) of the core genes were highly similar in the monomer and polymer treatments for a given compound class. The core in the carbohydrate treatments (mono- and polysaccharides) consisted of 204 shared genes (accounting for ∼36% of total reads) which reached a significantly higher relative abundance in the CH-core compared to the controls, making it the largest core. In comparison, as many as 458 genes (53%) were specifically overrepresented with monosaccharides and 251 genes (∼30%) were specific to polysaccharides (Figures 4A, 5A). Among the nucleic acid core (Nuc-core) genes, 184 genes (∼13%) were significantly more abundant compared to controls (Figures 4A, 5A). The number of genes specific to either the nucleotides or DNA treatments was fairly low at 71 and 11 genes, respectively (Figure 4B). With only 66 genes (∼3–4%), the protein core (PR-core) was the smallest of the three studied cores (Figure 4C). In contrast, as many as 381 genes (∼65%) were specific to amino acids and 301 genes (46%) to polypeptides (Figures 4C, 5C).

A comparison of the three core gene sets showed that only five genes with a significantly higher relative abundance in the treatments were shared between the three cores (Figure 4D). More genes were shared between the carbohydrate and the nucleic acid cores (71 genes) than with the protein core (only 5 genes). Only three genes were found in the intersect between the nucleic acids and the protein cores (Figure 4D). An additional comparison of the monomer- and polymer-non-cores separately showed that monomeric and polymeric non-cores were remarkably distinct in the set of shared genes across the three compound classes (Figures 4E,F).

### Core and Non-core Gene Expression Responses Characterizing the Different Compound Classes

A comparison of the compound-class core genes grouped into top-level metabolic SEED categories revealed pronounced differences in the metatranscriptomes associated with the three tested compound classes (Figure 5, Excel sheets in Supplementary Table S4). The carbohydrate core comprised numerous and highly expressed genes that were associated with motility and chemotaxis (∼14% of total reads), encoding for nearly complete bacterial flagellar complexes and chemotaxis proteins, but they made up a minor portion of the nucleic acid core (below 0.3%) and were absent from the protein core (Figures 5A,C). In the carbohydrate core, the categories *Protein Metabolism, Amino Acid* (up to ∼4% each) and *Carbohydrate Metabolism* (below 2%) were abundant, collectively pointing toward the importance of L-arginine degradation (e.g., NAD-specific glutamate dehydrogenase), glycogen metabolism (e.g., glucose-1-phosphate adenylyltransferase (∼0.2%, first rate-limiting step of glycogen biosynthesis), and utilization of labile carbon compounds (e.g., chitin N-acetylglucosamine, D-galacturonate, D-glucuronate, maltose, maltodextrin, deoxyribose, deoxynucleoside and trehalose, sucrose, xylose, rhamnose, and ribose). In the nucleic acid core, the SEED categories *Amino Acids* and *Carbohydrates* were abundant (∼2.5%), as illustrated by a nearly complete L-arginine succinyltransferase (AST) pathway. Rather surprising was the low abundance of *Membrane Transport* (∼0.1% of total reads) in the carbohydrate core, that accounted for ∼0.6% in the nucleic acid core (e.g., TonB transport systems and nucleoside permease NupC) and for up to ∼0.4% in the protein core (e.g., inorganic phosphate transporters). Both the nucleic acid and the carbohydrate cores showed a dominance of expressed genes (below 1%) in the *Vitamin* category that was relatively low in the protein core (∼0.3%) (Figures 5A,C).

**FIGURE 6:**
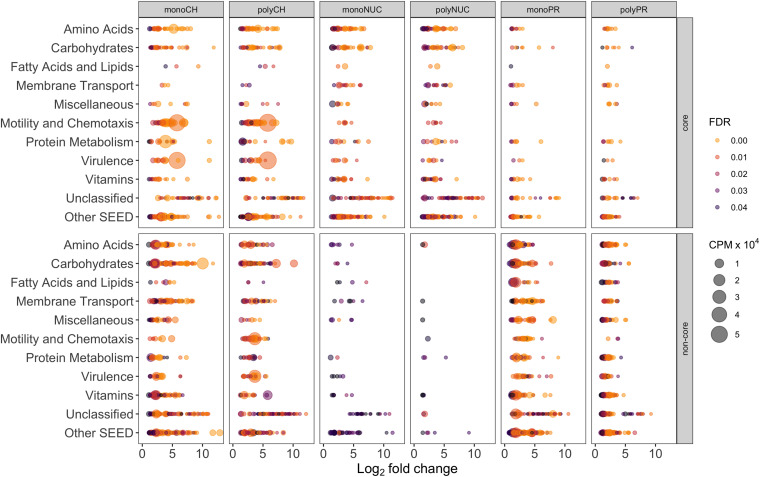
Individual gene-level expression of significantly overrepresented genes (positive log_2_-fold-change compared to controls) grouped into the 10 most abundant SEED categories. Genes with a false discovery rate (FDR) below 5% in treatments compared to controls are displayed. The bubble size corresponds to the mean (*n* = 3, except polyPR *n* = 2) of normalized counts per million (cpm × 10^4^) of total reads, the colorbar represents FDR values. Note that the numbers of genes are inflated due to multiple roles of certain genes, thus assignment to more than one SEED category is possible. Abbreviation of compound classes: CH, carbohydrates; NUC, nucleic acid; PR, proteins. Abbreviation of condensation states: mono, monomers; poly, polymers. “Core” refers to shared response in monomers and polymers for a given compound class; “non-core,” responses specific to a certain treatment excluding the core fraction.

To determine the potential influence of monomers compared to polymers on bacterioplankton community transcription, we analyzed the specific (i.e., non-core) genes with a significantly higher relative abundance in the different DOM treatments relative to controls (Figures 5B,D, Excel sheets in Supplementary Table S4). All non-cores (monomers and polymers) showed noticeable differences in the gene expression patterns between compound classes in a broad variety of SEED categories (Figures 5B,D). In the carbohydrate treatments, *Motility and Chemotaxis* differed substantially between poly- (∼6% of total reads) and monosaccharides (∼1%); for example, in the polysaccharides many Type IV pili associated genes (e.g., PilY/M/A/W/E) were highly expressed (Figures 5B,C). In addition to genes in the polysaccharide non-core that were related to carbohydrate metabolism (4%), e.g., malate synthase (∼0.9%) and malate synthase-related protein (∼0.5%), genes indicative of utilization of carbohydrates (e.g., glycogen, chitin, rhamnose, xylose), and degradation of polysaccharides (e.g., beta-glucosidase) were highly expressed. Categories with a higher relative abundance in the monosaccharide non-core were for example *Carbohydrates* (∼10%), *Vitamins* (6% compared to ∼2% in polysaccharides), *Amino Acid*s (∼6% compared to ∼3%). In particular, L-arginine and putrescine (synthesis and degradation) genes, and *Membrane Transport* (∼5% compared to ∼2%) that consisted of numerous genes relevant for the transport of branched-chain amino acids (0.4%), polyamines (∼0.3% e.g., PotD/F/A/G/B/C), and many ABC type transporters with specificity toward fructose (0.3%, e.g., FrcA/B/C), ribose (0.1% e.g., RbsA/B/C) and various polyols (i.e., selenate and selenite) (Figures 5, 6, and Supplementary Table S4), that were not found in the polysaccharide non-core. In the amino acid non-core, *Membrane Transport* (∼4%) was 4-fold more abundant, encoding amino acid and polyamine specific transporter genes (below 0.1% e.g., polyamine transport–PotA/B/C/H, histidine, and arginine ABC transporter) compared to the polypeptide non-core that comprised only a few transporter associated genes e.g., for sulfate, iron, and phosphate (e.g., PstC/B/A) (Figures 5, 6, and Supplementary Table S3). Additional functional features that were enriched in the amino acid non-core, found in the *Amino Acids* category, were e.g., parts of the ILV (isoleucine, leucine, and valine) degradation pathway, *Carbohydrates* (below ∼8% each), *Fatty Acids and Lipids* and *Motility and Chemotaxis* (∼2%) that consisted of many chemotaxis associated genes (32 genes, e.g., CheV/A/Y/Z/R/W/B/C), twitching motility and Type IV pili (Figures 5B,C). The gene expression in the nucleic acid non-core was very low (below ∼3%) compared to the other compound classes, but some categories differed notably between condensation states, for example, *Amino Acids.* Notably, in the nucleotides non-core, *Motility and Chemotaxis* was undetectable, whereas the *Carbohydrates* and *Fatty Acids and Lipids* categories were absent from the DNA non-core (Figures 5B,C).

### Functional Signature of Compound Classes and Condensation States

Lastly, a search for gene systems encoding ecologically relevant functions uncovered “functional signatures” that differed between compound classes and condensation states, both in the number of genes and relative abundance (Supplementary Figures S4–S6). For example, the carbohydrate core was enriched with transferases (14 genes accounting for ∼1.5% of total reads) compared to nucleic acid and protein cores (∼16 genes, ∼1% and 9 genes, ∼0.5% of total reads) (Supplementary Figures S4–S6). However, in the carbohydrate non-cores, the most striking functional signatures were associated with transporters (92 genes in the monosaccharide non-core but only 13 in the polysaccharide non-core, accounting for 4.1% and 0.2% of total reads, respectively) and transferases (42 genes ∼5% and 21 genes ∼2%). Similarly, the protein non-cores were enriched in transferases, 58 genes were detected in the polypeptide non-core and 30 in the amino acid non-core (accounting for ∼4% and ∼2% of total reads). Genes coding for reactions associated with carbohydrate-active enzymes (CAZymes) were primarily detected in the non-cores with the highest expression in the carbohydrates (21 genes, ∼1% of total reads) and protein non-cores (Supplementary Figure S4–S6).

## Discussion

Our study demonstrates how marine bacteria responded to two major facets of DOM characteristics. First, we found pronounced differences in bacterial growth, activity, and functional gene expression upon exposure to different compound classes (i.e., carbohydrates, nucleic acids, and proteins). Second, within the carbohydrates and protein compound classes (but not nucleic acids), bacterial responses differed between enrichments with monomers compared to polymers (i.e., condensation state). Thereby we substantially expand on the few studies that have directly compared bacterial responses to different labile polymers and their constituent monomers (Cottrell and Kirchman, 2000; Bryson et al., 2017), showing that resource partitioning between bacterial taxa is influenced by the condensation state of the DOC compounds. These considerations emphasize that analyses of bacterial growth and gene expression responses to complementary sets of chemically defined and ecologically relevant DOC compounds, differing for example in nutrient stoichiometry or condensation states, or both, allow gaining novel knowledge of the molecular mechanisms regulating the marine carbon cycle (Moran et al., 2016).

A first notable observation on the bacterial responses to the studied carbon compounds was the ample variability in growth dynamics between monomers and polymers both within and between compound classes (Figure 1). This links with the complex discussion regarding whether marine bacteria preferentially rely on low- or high-molecular-weight DOC for growth and how molecular weight influences lability and transformation rates of dissolved organic matter (Moran et al., 2016). In our experiments, bacteria responded quickly to enrichment with either carbohydrates or protein (and fairly slow to nucleic acids), and associated with the polymers in each of these two classes there were strong increases in glucosidase and aminopeptidase activities, respectively. Still, it was only in the protein enrichments that there were pronounced differences in growth, with higher yields in polypeptides compared to amino acids. In a previous study, we observed the fastest growth responses and highest cell yields in enrichments with amino acids compared to monomers like glucose (Gomez-Consarnau et al., 2012). In the current study though, the highest cell numbers were in the polypeptide treatment; currently, we cannot specify if this was due to smaller bacterial cell sizes or to a higher total biomass yield with polypeptides. Nevertheless, our findings on the utilization of monomers and polymers in distinct biopolymer classes, that are released into the water through various ecological processes, indicate that both low- and high-molecular-weight DOC can be considered labile or “user-friendly” from a bacterial community perspective.

Second, bacterial growth showed interestingly divergent linkages with overall gene expression responses. Thus, it could be expected that there would be little, if any, differences in gene expression between monomers and polymers of the same compound class when the growth responses in the two condensation states were highly similar—as was the case for nucleotides compared to DNA. Also, it was expected to find profound differences in gene expression when the growth patterns diverged substantially between monomers and the corresponding polymers–as for the amino acids compared to polypeptides. Unexpectedly, and importantly, the responses in the carbohydrate amendments showed that although the growth dynamics were highly similar in the treatments with simple sugars as compared to polysaccharides, the gene expression patterns differed substantially. Collectively, these divergent responses indicate that the manners in which bacteria deal with distinct carbon compounds are more complex than what would be inferred from the overall growth dynamics.

### Gene Expression Analyses

In our quest for disentangling the molecular mechanisms that regulate the complex interactions between marine bacteria and dissolved organic matter, it was important to note that the bacterial gene expression patterns were structured at several levels. This included: (i) significant effects on functional gene expression of DOM at the level of compound classes (i.e., distinguishing responses to carbohydrates, proteins, and nucleic acids) and also (ii) at the level of particular monomers and polymers (i.e., in the cases of mono- vs. polysaccharides and amino acids vs. polypeptides, but marginally for DNA vs. nucleotides). Moreover, (iii) there were pronounced taxonomic differences in the expression responses to the different carbon compounds. In the following, we will discuss key features of these three points, and their interdependencies toward selectively shaping bacterial expression responses.

#### Core Gene Responses

The finding of sets of expressed genes that differed significantly between the compound classes carbohydrates, proteins, and nucleic acids (but were shared between monomer and polymer enrichments within each of the classes) indicated the response of “core genes” characteristic to the compound classes. These core gene responses were different already at the level of broad functional categories (i.e., top-level SEED categories), which emphasizes the pronounced influence the availability of resources has on bacterial metabolism. In several cases, the core gene sets matched expectations concerning the added DOM. Accordingly, the carbohydrates core showed several-fold higher expression of glucosidases relative to controls (most pronounced in polysaccharides). Moreover, genes involved in the biosynthesis of glycogen were significantly more abundant in the carbohydrates core (including the gene for glucose-1-phosphate adenylyltransferase–the enzyme that catalyzes the rate-limiting first-step in the biosynthesis of glycogen). Indeed, investment in metabolic storage compounds such as glycogen is an important strategy to cope with dynamic changes in nutrient availability (Wilson et al., 2010).

More surprising was the high expression of motility and chemotaxis genes in the carbohydrates core not seen in the other cores, suggesting that compound class may be more important than the condensation state in inducing responses of motile and chemotactic bacteria. This motility response was observed in both the mono- and polysaccharide treatments even though the expression in these treatments was dominated by different taxa (and even though Alteromonadales largely dominated also in the nucleic acids treatments, their expression of motility genes there was ∼5-fold lower). It has been noted that motility is often coupled with the expression of hydrolyzing enzymes and that motility and chemotaxis play a paramount role in the degradation of organic matter in the sea (Blackburn et al., 1998; Stocker, 2012). While there is knowledge on the nature of chemoattractants (e.g., amino acids, organic acids, carbohydrates) in bacteria (Pandey and Jain, 2002; Kalinin et al., 2010; Fernandez et al., 2019), the role that the condensation state has in triggering gene expression responses in motile and chemotactic marine bacteria warrants further research. Also, curious, the carbohydrates core consisted of a nearly complete L-arginine catabolism pathway. A review by Cunin et al. (1986) reported that few gram-negative proteobacteria use the arginine succinyltransferase (AST) pathway to utilize L-arginine as the sole carbon and nitrogen source. Yet, it remains unclear what induced this particular pathway in the carbohydrate treatments.

As could be anticipated, the nucleic acids core was enriched in genes involved in DNA catabolism and conversion, encoding enzymes for the utilization of purine and pyrimidines that allow active usage of the added nucleic acids as carbon, nitrogen, and phosphorus sources (Kirchman, 2003). The expression responses in the protein core were characterized by genes associated with amino acid metabolism but also with phosphorus metabolism. The latter involved genes for both uptake and hydrolysis of phosphate compounds is interesting in that it points to a demand for P to obtain a balanced stoichiometry in face of the high content of C and N in protein (amino acids and polypeptides alike). Collectively, these findings indicate that the chemical properties inherent to different DOM compound classes, for instance, the variability in elemental ratios, chemical linkage types, and chemical bonds, exert differential selection pressure on bacterial gene expression.

#### Monomer- and Polymer-Specific (Non-core) Gene Expression Responses

In the following, we discuss the patterns in the expression of functional genes in the monomer and polymer treatments in the context of the expression by different bacterial groups since they were tightly linked. Interestingly, these specific monomer/polymer responses were larger than the compound class-wide set of core genes (both in number of genes and relative expression). The monosaccharide-specific gene set (non-core) consisted of a noteworthy number of transporter associated genes (Supplementary Figure S2), out of which many were associated with monosaccharide uptake as could be anticipated. Rather surprising though, was the presence of several genes encoding transporters that are specific for polyols, nucleosides, amino acids, and polyamines. Potentially, the availability of monosaccharides may have provided additional energy that induced the acquisition of compounds that were present in the original seawater. Members of the Oceanospirillales that dominated the expression in the monosaccharide treatments are known as fast responders to oil spills thanks to near-complete pathways for non-gaseous n-alkane and cycloalkane degradation (Mason et al., 2012, 2014). Also, genomic analyses of uncultured Oceanospirillales representatives found genes associated with chemotaxis and motility capabilities besides a complete B12 vitamin synthesis pathway in conjunction with a suite of transporters for scavenging nutrients such as amino acids, fatty acids, carboxylic acids, ammonium, iron, sulfate, and phosphate (Mason et al., 2012; Delmont et al., 2015). In addition, *Marinomonas* sp. is taking part in the ocean sulfur cycle through catabolism of dimethylsulfoniopropionate (DMSP) resulting in the formation of dimethyl sulfide (DMS) (Todd et al., 2007). Our experimental analysis showed that Oceanospirillales are extraordinarily efficient in scavenging certain monomers, especially monosaccharides, thereby adding an important ecological feature to this bacterial group.

The most striking feature of the polysaccharide non-core was the pronounced expression of genes encoding motility and chemotaxis, in addition to type IV pili genes. Type IV pili can have a multitude of functions (e.g., adherence and aggregation, motility, biofilm formation, competence and conjugation, protein secretion) (Chen and Dubnau, 2004; Giltner et al., 2012; Maier and Wong, 2015), and can be involved in inter-bacterial interactions during surface colonization of the polymer chitin (Adams et al., 2019). The dominant expression of Alteromonadales (*Colwellia* sp. and *Shewanella* sp.) in the polysaccharide treatment is in accordance with their recognized ability to utilize labile DOC and of outcompeting other bacteria under high nutrient concentration (McCarren et al., 2010; Sarmento and Gasol, 2012; Pedler et al., 2014). These opportunists can comprise significant proportions of natural bacterioplankton communities both in terms of abundance and activity (Acinas et al., 1999; Teeling et al., 2012). The genomic predisposition toward a diverse set of organic matter compounds, together with the experimental evidence for responsiveness to polysaccharides and nucleic acids provided here, thus make important parts of the “feast-and-famine” lifestyle of Alteromonadales (McCarren et al., 2010; Pedler et al., 2014; Koch et al., 2020).

Also, members of the order Chromatiales contributed to the expression in the polysaccharide treatment with strong expression signals in carbohydrate and amino acid metabolism. Whereas this order encompasses well-known purple sulfur bacteria that are typically photoautotrophs with a limited ability of photoheterotrophy, it also contains typically heterotrophic bacteria such as the genus *Rheinh*eimera that was transcriptionally active in our experiments and that is found in diverse aquatic environments (Brettar et al., 2002; Chen et al., 2010). In Baltic Sea experiments, *Rheinheimera* sp. were highly responsive to nutrient additions (e.g., glucose, ammonium, phosphate, n-alkanes) or changes in environmental conditions (Pinhassi and Berman, 2003; Lindh et al., 2015a; Karlsson et al., 2019). A search for *Rheinheimera* sp. in the carbohydrate-active enzyme (CAZy) database (http://www.cazy.org; last update on June 26, 2020) showed that at least two genomes (*Rheinheimera* sp. D18 and F8) contain a notable number of glycoside hydrolase families (e.g., GH1, GH3, GH13, GH23, GH73, GH77, and GH103), and other carbohydrate-degrading enzymes (data not shown). These findings suggest that Chromatiales and *Rheinheimera* related taxa in particular may be important in the turnover of polymers.

Rather unsurprisingly, amendments with amino acids resulted in an overrepresentation of expressed genes in amino acid metabolism (but distinct from amino acids metabolism in the polypeptide treatment, see below) and an outer membrane porin (OprD) with known specificity for cationic amino acids and peptides (Tamber et al., 2006). The expression of polyamine related transporters was curious. These polycationic compounds (e.g., putrescine and spermidine), which are produced in large quantities during phytoplankton blooms in the sea (Nishibori et al., 2001), are rich in carbon and nitrogen and are required for the synthesis of deoxyribonucleic acids (DNA), ribonucleic acids (RNA) and proteins (Tabor and Tabor, 1985; Leigh and Dodsworth, 2007). Amendments with amino acids resulted in a functional dominance of Pseudomonadales (in particular *Pseudomonas*). The genus *Pseudomonas* (Gammaproteobacteria) is extremely diverse in regard to metabolism, physiology, and genetics (Palleroni and Doudoroff, 1972; Spiers et al., 2000) and includes pathogens as well as species efficient in the degradation of toxic compounds and aromatic hydrocarbons (Bartolome-Martin et al., 2004; Teufel et al., 2010; Wasi et al., 2013). Griffith and Fletcher (1991) measured high hydrolysis rates of dipeptides and proteins in incubations with *Pseudomonas* isolates, both in solution and attached to diatom particles (Griffith and Fletcher, 1991). Moreover, many gammaproteobacteria, including *Pseudomonas*, are able to utilize branched-chain amino acids as sole carbon and energy sources via the ILV (i.e., isoleucine, leucine, and valine) degradation pathways (observed here) (Kazakov et al., 2009). These findings highlight the potential linkage between polyamines and amino acids (especially branched-chain amino acids) and the ability of Pseudomonadales (i.e., *Pseudomonas* and *Psychrobacter*) to outperform other bacteria in the quest for these labile organic monomers.

In the polypeptide treatment, genes in the SEED categories *Protein Metabolism* and *Amino Acids* were highly expressed (but subcategories in amino acid metabolism differed as compared to the treatment with amino acids added as monomers). Another interesting observation was the relatively high abundance of transferases, especially these targeting phosphorus-containing groups (Supplementary Figures S4, S5), whereby bacteria synthesize important macromolecules (Lairson et al., 2008). Flavobacteriales dominated virtually all metabolic subcategories in the polypeptide amendments. The Flavobacteriales (Bacteroidetes) are recognized as exceptionally efficient polysaccharide degraders, both in laboratory experiments and field observations (Pinhassi et al., 2004; Teeling et al., 2012; Fernandez-Gomez et al., 2013; Bennke et al., 2016). Accordingly, comprehensive genome analyses of Flavobacteriia reveal the presence of multiple polysaccharide utilization loci (PULs) with important ecological implications (Fernandez-Gomez et al., 2013; Grondin et al., 2017; Kappelmann et al., 2019). Noteworthy, multiple reports emphasize that Bacteroidetes encode numerous peptidases that can even outnumber glycoside hydrolases (GHs) (Fernandez-Gomez et al., 2013; Mann et al., 2013). An early field study using microautoradiography demonstrated a preference of Bacteroidetes for proteins over amino acids, with roughly half of the cells utilizing protein but only a minor fraction consuming amino acids (Cottrell and Kirchman, 2000). Orsi et al. (2016) expanded on this observation reporting a substantial contribution of particle-attached Flavobacteria to the utilization of proteins in the upper coastal Pacific Ocean (Orsi et al., 2016). Possibly, a preference for protein-rich compounds over free amino acids could be driven by the fact that peptides provide more carbon, and ultimately more energy, per nanomole at a similar energetic cost compared to the same concentration of dissolved free amino acids (Kirchman and Hodson, 1984; Casey et al., 2015). Collectively, these results further suggest that, besides the recognized importance of Flavobacteria in the hydrolysis of carbohydrates, they play an important role in the transformation of proteins in seawater (Pinhassi et al., 1999; Fernandez-Gomez et al., 2013).

#### Influence of Monomers and Polymers on Selective Divergence in Taxon-Specific Expression

A principle question raised by our finding is what determined the strong divergence in functional gene expression responses by distinct taxa in the monomer and polymer treatments within the carbohydrates and protein compound classes (i.e., non-core responses), even though the polymers consist of the same monomers. A harmonious explanation for this observation would be that populations that dominate the polymer treatments do so by being better at hydrolyzing polymers (Baltar, 2017), whereas monomer specialists are more successful in the uptake of monomers (Alonso and Pernthaler, 2006). Indeed, in the polymer treatments, we measured very high hydrolysis rates for polysaccharides and proteins (glucosidases and aminopeptidases, respectively) (Supplementary Figure S2A), and there were tendencies toward a higher expression of hydrolytic enzyme genes in the polymer treatments and of membrane transporters in the monomer treatments. Given that, ultimately, the compounds transported into the cell are essentially the same monomers (although substrates could also be transported as oligomers), irrespectively of whether initially provided as polymers or monomers, our results suggest that the “first encounter” (e.g., sensing, assimilation, consumption, affinity) of the different compounds exerts a very strong selection for expression by different taxa. In this scheme, the “first encounter” essentially would consist of hydrolytic enzymes and membrane transporters.

Our results extend on the elegant phrasing by Arnosti et al. (2011) that “enzymatic hydrolysis is the initial step leading of the entire remineralization cascade”, so that the initial hydrolysis and subsequently uptake across the cell membrane apparently orchestrates remodeling of the gene expression repertoire of bacterial assemblages. Such changes in expression are likely to have downstream effects, influencing which additional and complementary metabolic pathways that are induced depending on the successful taxa. Shifts in expression would be further accentuated by changes in the abundance of different bacterial populations (Aylward et al., 2015) and the sets of constitutively expressed metabolisms among increasing populations. These findings imply that differences in DOM composition, and in this context the condensation state of compounds, would influence successional patterns by favoring bacteria differing in a gradient from hydrolyzer to monomer specialists.

A concern regarding our interpretation of bacterial responses to different DOM components of different compound classes and condensation states could be that we carried out two experiments each using water collected in February and mid-March, respectively, and that the two experiments tested different sets of DOM compounds. Since bacterial community composition changes over time, differences in the inoculum composition could affect the results by influencing which bacteria became dominant in a given treatment. We did not determine the community composition at the DNA level (16S rRNA gene analysis), and therefore can not directly evaluate the potential influence of the composition of the inoculum. Indeed, analysis of the gene expression responses indicated differences in taxonomic groups in the controls of the two experiments. For example, the Pelagibacterales were only transcriptionally active in experiment 2. Nevertheless, the presence of specific bacterial groups in each of the controls did not necessarily correspond to the groups dominating the expression in the treatments of the same experiment. For instance, Pseudomonadales made up a larger proportion of the expression in the controls from experiment 1, yet they dominated the polypeptide treatment that was tested in experiment 2. Collectively, we think that these observations indicate that the effect of differences in the starting communities was smaller relative to the influence of the DOM compound characteristics.

It has been shown that different DOC compounds select for specific sets of bacterial populations and often for distinct genetic functions (Cottrell and Kirchman, 2000; Mou et al., 2008; Gomez-Consarnau et al., 2012; Gifford et al., 2013). Going into further detail on uncovering the mechanisms of bacterial foraging strategies of potential importance in the wild, a few studies investigated bacterial growth, uptake, and enzymatic activity responses to different sets of *a priori* selected model compounds that differ in condensation state (i.e., monomers–e.g., glucose, amino acids, and N-acetyl-D-glucosamine compared to polymers–e.g., starch, protein, chitin, and oligopeptides) (Cottrell and Kirchman, 2000; Riemann and Azam, 2002; Obayashi and Suzuki, 2008; Bryson et al., 2017). Collectively, these studies showed that the utilization of low- and high-molecular-weight DOM compounds differed across broad phylogenetic groups, suggesting a high degree of specialization and resource partitioning among distinct bacteria. Our results substantially expand on these findings by providing detail on the functional genes that potentially account for resource partitioning at a fine-scale between multiple sets of monomers and polymers. This opens the possibility in the near future to explore the interdependence between particular DOM components and bacterial community composition. In particular, it would be important to uncover how successional patterns of marine bacteria are regulated by selection on particular genomic traits as realized through gene expression.

## Conclusion

Our analysis uncovered pronounced differences in bacterial growth and gene expression responses when supplied with ecologically relevant DOM compound classes (including carbohydrates, nucleic acids, and proteins) and in two distinct condensation states (i.e., monomers and polymers). The systematic differences in gene expression profiles depending on DOM characteristics emphasized that: (i) the two facets of DOM quality–i.e., compound class and condensation state–influence bacteria in complementary ways, and (ii) DOM composition is a major driving force in structuring the functional responses of specific key bacterial taxa.

We think these two points emerged largely thanks to that transcriptional responses differed between compound classes, showing “core gene” responses for carbohydrates (notably genes for labile carbon compound utilization, glucosidases, and motility and chemotaxis), proteins (e.g., amino acids and phosphorus metabolism genes), and nucleic acids (e.g., DNA conversion, nucleotides utilization, and TonB transporters). In addition, there were important taxon-specific responses that included membrane transporters (Oceanospirillales; in monosaccharides treatment), motility and glycogen utilization (Alteromonadales; in polysaccharides), uptake systems for amino acids and polyamines (Pseudomonadales; in amino acids), and transferases and uptake systems (Flavobacteriales; in polypeptides). Responses specific to DNA and nucleotides were both dominated by Alteromonadales.

As a consequence, the specialization of bacterial populations to utilize polymers in comparison to monomers can be a factor decisive for regulating bacterial population dynamics in response to upwelling-driven phytoplankton blooms and seasonal succession. These findings showcase the interdependency between the genomic complement of marine bacteria and the precise architecture of labile DOM components that collectively regulates the “invisible” cycling of labile DOM in surface waters of the ocean.

## Data Availability Statement

The metatranscriptome sequence dataset generated in this study is deposited in the EMBL-EBI European Nucleotide Archive repository (https://www.ebi.ac.uk/ena), under the primary accession PRJEB32140.

## Author Contributions

BP and JP designed the study. BP conducted the experiments, processed, collected the data, and wrote the first draft of the manuscript. BP and DL analyzed the data. All authors interpreted the data and contributed to subsequent revisions.

## Conflict of Interest

The authors declare that the research was conducted in the absence of any commercial or financial relationships that could be construed as a potential conflict of interest.
