# Supplementary material for: Labile Dissolved Organic Matter Compound Characteristics Select for Divergence in Marine Bacterial Activity and Transcription
Source: Front Microbiol. 2020 Sep 25;11:588778. doi: 10.3389/fmicb.2020.588778 (PMC7546218; doi:10.3389/fmicb.2020.588778)
Supplement: Supplementary file 2 [file Data_Sheet_1.docx]

Supplementary Material

# Supplementary Figures and Tables

**Supplementary Figure S1.** Location of the Linnaeus Microbial Observatory (LMO) time-series station (Coordinates: N 56°, 55.8540', E 17°, 3.6420') in the Baltic Sea. The station is located ~10 km offshore from the east coast of Öland (Sweden). The color code depicts the sea surface temperature (SST) from Multi-scale Ultra-high Resolution (MUR) satellite measurements that were obtained from the National Oceanic and Atmospheric Administration (NOAA) ERDDAP during the time of sampling on February 16th, 2016.

**Supplementary Figure S2.** Enzymatic activity (EEA) measurements of four different substrate analogues. Panels **(A-D)** show the mean hydrolysis rate of tested enzymes of biological replicates. Panels **(E-H)** depicting normalized hydrolysis rates per cell. Abbreviation of substrate analogues: AGase, 𝛼-glucosidase (**A** and **E**); BGase, 𝛽-glucosidase (**B** and **F**); LAPase, leucine aminopeptidase (**C** and **G**); APase, alkaline phosphatase (**D** and **H**). Abbreviations of treatments: monoCH, monosaccharide mix (n = 3); polyCH, polysaccharide mix (n = 3); monoNUC, nucleotide mix (n = 3); polyNUC, DNA (n = 3); contOne, untreated seawater from experiment 1 (n = 3); monoPR, amino acid mix (n = 3); polyPR, polypeptides (n = 3); and contTwo, untreated seawater from experiment 2 (n = 3); Cbx, carboxylic acids (n = 3).

**Supplementary Figure S3.** Bacterial production (BP) of the nutrient enrichment experiments. Panels (**A** and **B**) show the bacterial production of the total community, (**C** and **D**) show the mean bacterial production normalized per cell. Abbreviations of treatments: monoCH, monosaccharide mix (n = 3); polyCH, polysaccharide mix (n = 3); monoNUC, nucleotide mix (n = 3); polyNUC, DNA (n = 3); contOne, untreated seawater from experiment 1 (n = 3); monoPR, amino acid mix (n = 3); polyPR, polypeptides (n = 3); contTwo, untreated seawater from experiment 2 (n = 3); Cbx, carboxylic acids (n = 3). Panels (**A** and **C**) show the measurements from experiment 1 (E1), whereas (**B** and **D**) from experiment 2 (E2).

**Supplementary Figure S4.** Overview of the “functional signature” of compound classes and condensation states divided into core and non-core. Shown are relative abundances and numbers of genes, with significantly higher relative abundances in treatments relative to controls, grouped into CAZymes, peptidases (EC 3.4 and 6.3), transferases (EC 2.1 - 2.10), and transporters. Relative abundances are denoted as the mean of normalized counts per million (cpm x 10^4^) of biological replicates (all treatments n = 3, except polyPR n = 2). The numbers on top of the bars depict the number of genes found in each group. Abbreviation of compound classes: CH, carbohydrates; NUC, nucleic acid; PR, proteins. Abbreviation of condensation states: mono, monomers; poly, polymers. Core, shared response in monomers and polymers for a given compound class; non-core, responses specific to a certain treatment excluding the core fraction.

**Supplementary Figure S5.** Detailed information of the “functional signature” of compound classes and condensation states showing CAZyme classes (EC numbers associated with glycoside hydrolases, glycosyltransferases, carbohydrate esterases, polysaccharide lyases, auxiliary activities, carbohydrate- binding-modules), peptidases (EC 3.4 and 6.3), transferases (EC 2.1 - 2.10), and transporter families. Depicted are mean relative abundances of selected genes based on a false discovery rate (FDR) of 5% relative to controls (all treatments n = 3, except polyPR n = 2). Abbreviation of compound classes: CH, carbohydrates; NUC, nucleic acid; PR, proteins. Abbreviation of condensation states: mono, monomers; poly, polymers. “Core” refers to shared response in monomers and polymers for a given compound class; “non-core” to responses specific to a certain treatment excluding the core fraction. Abbreviation of transporter families: CUT1 and CUT2 - Carbohydrate uptake transporter 1 and 2, respectively; POPT - Polyamine/Opine/Phosphonate Uptake Transporter; QAT - Quaternary Amine Uptake Transporter; FECT - Iron Chelate Uptake Transporter; PAAT - Polar Amino Acid Uptake Transporter; HAAT - Hydrophobic Amino Acid Uptake Transporter; PEPT - Peptide/Opine/Nickel Uptake Transporter; PHOT - Phosphate Uptake Transporter; MOLT - Molybdate Uptake Transporter; PHNT - Phosphonate Uptake Transporter; No TC - transporter associated genes without a transporter classification number (TC). Note that the total number of CAZymes is larger than shown in Supplementary Figure S4 due to the assignment of certain enzymes into multiple CAZyme classes.

**Supplementary Figure S6.** Gene-level information of “functional signatures” of compound classes and condensation states showing CAZyme classes (EC numbers associated with glycoside hydrolases, glycosyltransferases, carbohydrate esterases, polysaccharide lyases, auxiliary activities, carbohydrate- binding-modules), peptidases (EC 3.4 and 6.3), transferases (EC 2.1 - 2.10), and transporters. Depicted are mean relative abundances (all treatments n = 3, except polyPR n = 2) and log2 fold change of selected genes based on a false discovery rate (FDR) of 5% relative to controls. Abbreviation of compound classes: CH, carbohydrates; NUC, nucleic acid; PR, proteins. Abbreviation of condensation states: mono, monomers; poly, polymers. “Core” refers to the shared response in monomers and polymers for a given compound class; “non-core”, responses specific to a certain treatment excluding the core fraction. Abbreviation of transporter families: CUT1 and CUT2 - Carbohydrate uptake transporter 1 and 2, respectively; POPT - Polyamine/Opine/Phosphonate Uptake Transporter; QAT - Quaternary Amine Uptake Transporter; FECT - Iron Chelate Uptake Transporter; PAAT - Polar Amino Acid Uptake Transporter; HAAT - Hydrophobic Amino Acid Uptake Transporter; PEPT - Peptide/Opine/Nickel Uptake Transporter; PHOT - Phosphate Uptake Transporter; MOLT - Molybdate Uptake Transporter; PHNT - Phosphonate Uptake Transporter; No TC - transporter associated genes without a transporter classification number (TC). Note that the total number of CAZymes is larger than shown in Supplementary Figure S4 due to the assignment of certain enzymes into multiple CAZyme classes.

| **Supplementary Table S1.** Bacterial cell counts (n = 2) of unfiltered *in situ* water and 0.6 µm pre-filtered water. | | | |
| --- | --- | --- | --- |
| **Experiment** | **Cells mL^-1^ x 10^6^ (unfiltered)** | **Cells mL^-1^ x 10^6^ (0.6 µm pre-filtered)** | **Remaining (%)** |
| E1 | 0.65 | 0.53 | 81.77 |
| E2 | 1.34 | 1.01 | 75.34 |
| Note: The pre-filtered fraction was used for bacterial inocula in seawater culture regrowth experiments. Samples were taken on February 16th for experiment 1 and on March 31st, 2016 for experiment 2 shortly before the start of each experiment (see also Material and Methods). | | | |

| **Supplementary Table S2.** DOC concentrations in the enrichment experiments and controls were measured immediately after the addition of compounds on day 0 for experiments 1 and 2. | | | |
| --- | --- | --- | --- |
| **Experiment** | **Compound class** | **Treatment** | **DOC (µM) ± SD (nr)** |
| 1 | Carbohydrates (CH) | Monosaccharides (monoCH)  Polysaccharides (polyCH) | 332 ± 2 (n = 3)  323 ± 3 (n = 3) |
|  | Nucleic acids (NUC) | Nucleotides (monoNuc)  DNA (polyNUC) | 317 ± 3 (n = 3)  324 ± 2 (n = 3) |
|  | Control | ControlOne | 313 ± 10 (n = 3) |
| 2 | Protein (PR) | Amino acids (monoPR)  Bovine serum albumin (polyPR) | 318 ± 2 (n = 2)  322 ± 11 (n = 3)* |
|  | Carboxylic acids (Cbx) | Carboxylic acids (Cbx) | 303 ± 2 (n = 3)* |
|  | Control | ControlTwo | 309 ± 11 (n = 3) |
| Values marked with asterisks should be interpreted with caution due to technical issues with the instrument during the analysis. | | | |

| **Supplementary Table S3.** Mean and standard deviation (± SD) of raw read counts (x 10^6^) for each treatment, merged paired-end reads (Assembled), functional (Seed), and taxonomic annotation (Taxonomy) in nutrient enrichments and controls. | | | | | | |
| --- | --- | --- | --- | --- | --- | --- |
| **Experiment** | **Compound class** | **Treatment** | **Raw** | **Assembled** | **Seed** | **Taxonomy** |
| 1 | Carbohydrates (CH) | monoCH | 29.3 ± 2.6 | 10.8 ± 2 | 2.3 ± 0.8 | 5.1 ± 1.4 |
|  |  | polyCH | 30.8 ± 1.2 | 4.7 ± 1.5 | 1.3 ± 0.4 | 2.5 ± 0.7 |
|  | Nucleic acids (NUC) | monoNUC | 33.8 ± 1.8 | 12.5 ± 2 | 3.9 ± 0.6 | 6.6 ± 1 |
|  |  | polyNUC | 34.7 ± 0.9 | 14.9 ± 2.7 | 5.3 ± 1.4 | 8.9 ± 1.5 |
|  | Control | contOne | 36 ± 3.1 | 18.2 ± 0.1 | 3.5 ± 0.2 | 6.5 ± 0.4 |
| 2 | Protein (PR) | monoPR | 34.3 ± 2 | 9.9 ± 3.6 | 1.6 ± 0.7 | 3.5 ± 1.5 |
|  |  | polyPR | 32.5 ± 6.6 | 23.6 ± 10.9 | 5.9 ± 3.4 | 12.6 ± 8 |
|  | Carboxylic acids (Cbx) | Cbx | 60 ± 35.5 | 27.1 ± 20.7 | 4.1 ± 3.5 | 9.2 ± 6.9 |
|  | Control | contTwo | 30.4 ± 4 | 16.1 ± 13.1 | 3.6 ± 3.5 | 7.9 ± 6.7 |
|  |  | **All treatments** | **35.1 ± 11.2** | **14.3 ± 8.7** | **3.3 ± 2.1** | **6.6 ± 4** |
| Abbreviation of treatment names: monoCH, monosaccharide mix (n = 3); polyCH, polysaccharide mix (n = 3); monoNUC, nucleotide mix (n = 3); polyNUC, DNA (n = 3); contOne, untreated seawater from experiment 1 (n = 2); monoPR, amino acid mix (n = 3); polyPR, polypeptides (n = 2); Cbx, carboxylic acids (n = 2); contTwo, untreated seawater from experiment 2 (n = 3); mean of all treatments (n = 9). | | | | | | |

**Supplementary Table S4.** Excel sheets depicting the full list of core and non-core gene expression responses covering top-level SEED categories (seed1), subcategories (seed2), and the gene-level (seed3). *Motility and Chemotaxis* made up a large proportion of the carbohydrate core, consisting of several genes representing nearly complete parts of bacterial flagellar complexes (e.g. FlaA/G, FlgL/K/F/B, FlgF/B, FliD/Q/S/L, and FlhF) and chemotaxis proteins (e.g. MotA/B). As anticipated, the polysaccharide non-core (6% of reads, 21 genes e.g. FlaB/D, FleQ/N, and FlgE/G/D/C/H) showed a stronger signal than the monosaccharide counterpart (1%, e.g. FliM, FlhB, and FliP). In addition, many Type IV pili associated genes (e.g. PilY/M/A/W/E) were highly expressed in the polysaccharide non-core. In comparison, these features were very low expressed in the nucleic acid core (below ~0.3 of reads e.g. PilA/W/E, FliL and FliO). While the protein core did not comprise any of those genes, the amino acid non-core consisted of many chemotaxis associated genes (~2% of total reads, 32 genes, e.g. CheV/A/Y/Z/R/W/B/C), twitching motility and Type IV pili (e.g. PilQ/T/M/C/O/B/H/N/Y/P/A/R/E/W/S), which were substantially lower expressed in the polypeptides. *Membrane Transport* reached the highest relative abundance in the nucleic acid core (~0.6% of total reads, e.g. TonB transport systems, and nucleoside permease NupC). The non-cores comprised only seven genes, of which the majority were found in the nucleotides non-core (below 0.1% of reads, e.g. nitrate/nitrite transporter, general secretion pathway protein F/I). The protein core (~0.4%) consisted of a few genes associated with inorganic phosphate transporters. As anticipated, the amino acids non-core depicted an overabundance of amino acid and polyamine specific transporter genes (below 0.1% e.g. leucine-specific transport system, LivK, histidine ABC transporter, HisP, putrescine transport ATP-binding protein PotA/B), whereas a more diverse set of transporters for sulfate, iron, and phosphate (e.g. PstC/B/A) was found in the polypeptide counterpart. Rather surprising was the low abundance (~0.1% of total reads) of transporters in the CH-core. Strikingly, however, the monosaccharide non- core (4.7% of total reads) consisted of numerous genes relevant for the transport of branched-chain amino acids (0.4% of total reads, 3.2-fold), polyamines (~0.3% of reads e.g. PotD/F/A/G/B/C), and many ABC type transporters with specificity toward fructose (0.3%, e.g. FrcA/B/C), ribose (0.1% e.g. RbsA/B/C) and various polyols (i.e. selenate and selenite), that were not found in the polysaccharide non-core. The carbohydrate core showed enrichments in the *Amino Acid* SEED category (~2.5% of total reads) related to the degradation of L-arginine e.g. the key enzyme NAD- specific glutamate dehydrogenase (up to 1.2% of total reads), providing ammonia (NH4) and 2- oxoglutarate. In addition to e.g. arginine N-succinyltransferase, succinylarginine dihydrolase, succinylglutamic semialdehyde dehydrogenase, and succinylglutamate desuccinylase. The importance of L-arginine and putrescine (synthesis and degradation) was found in the polysaccharide non-core (e.g. arginine decarboxylase, gamma-glutamyl-aminobutyraldehyde dehydrogenase, gamma- glutamyl-putrescine synthetase, gamma-glutamyl-GABA hydrolase, and gamma- aminobutyrate:alpha-ketoglutarate aminotransferase (below 0.1% of reads). The L-arginine succinyltransferase (AST) pathway was enriched in the nucleic acid core (up to 0.1% of total reads, e.g. arginine N-succinyltransferase, succinylglutamic semialdehyde dehydrogenase and succinylglutamate desuccinylase). Overrepresented in the nucleotide non-core (up to 0.1% of reads) were acetylglutamate kinase (second step in L-ornithine biosynthesis from L-glutamate), serine acetyltransferase (first reaction in L-cysteine biosynthesis from L-serine), and beta-ureidopropionase (uracil degradation pathway to generate beta-alanine and ammonium). The protein core consisted of many genes associated with the amino acid glutamate. For instance, we noticed high expression of delta-1-pyrroline-5-carboxylate dehydrogenase (irreversibly oxidizes glutamate-gamma- semialdehyde to glutamate in the proline degradation pathway, thus nitrogen producing), in addition to high glutaminase activity (catalyzes the reaction L-glutamine to L-glutamate + NH_3_) and glutamate racemase expression (converts L-glutamate into D-glutamate). Outstanding features in the amino acid non-core were related to isoleucine degradation (up to 0.1% of reads) such as 3-hydroxyacyl-CoA dehydrogenase, 3-ketoacyl-CoA thiolase, acyl-CoA dehydrogenase. Significantly enriched in the polypeptide non-core were a few dehydrogenase associated genes (below 0.1%), involved in the histidine metabolism (e.g. glycine, glutamate, leucine, saccharopine and shikimate, and urocanate hydratase). *Carbohydrate metabolism* affiliated genomic features in the carbohydrate (below 1.6% of total reads) were glycogen metabolism (e.g. glucose-1-phosphate adenylyltransferase (~0.2%, the first rate-limiting step of glycogen biosynthesis), and related to the utilization of labile carbon compounds (e.g. chitin N-acetylglucosamine, D-galacturonate, D-glucuronate, maltose, maltodextrin, deoxyribose, deoxynucleoside and trehalose, sucrose, xylose, rhamnose, and ribose). Genes with strong enrichments in the monosaccharide non-core were associated with carbon metabolism (up to 2% of total reads), e.g. phosphoenolpyruvate synthase (involved in the reductive TCA cycle and required for the synthesis of pyruvate in glycolysis and gluconeogenesis), malate synthase G (part in the glyoxylate cycle, irreversible condensation of acetyl-CoA with glyoxylate to form malate, bypassing steps that lead to a loss of CO2, thus responsible for almost all of the malate synthase activity in cells metabolizing glyoxylate that is formed during growth on glycolate), electron transfer flavoproteins A and B (serve as specific electron acceptors for dehydrogenases), acetyl CoA synthetase (involved in many pathways including synthesis of amino acid and degradation of ethanol and chitin), glucose-6-phosphate 1-dehydrogenase (first step in the Entner-Doudoroff Pathway), and glucokinase (last step in glycogen degradation). High expression levels specific to the polysaccharide treatment were e.g. malate synthase (~0.9%) and malate synthase-related protein (~0.5%) and related to the utilization of carbohydrates (e.g. glycogen, chitin, rhamnose, xylose), and degradation of polysaccharides (e.g. beta-glucosidase). The nucleic acid core showed strong signals related to central metabolism e.g. aldehyde dehydrogenase A (~0.5% of total reads) aerobically oxidizes L- lactaldehyde in two steps to pyruvate, malate dehydrogenase, and acetoacetyl-CoA. In addition, many genes (up to 0.4%) were related to the catabolism of deoxyribose, and deoxynucleosides i.e. purine nucleoside phosphorylase, thymidine phosphorylase, phosphopentomutase, and deoxyribose- phosphate aldolase. The nucleic acid non-cores were very small due to the large similarity in expression between nucleotide and DNA treatments. However, the protein core was composed of genes such as putative deoxyribonuclease, phosphate acetyltransferase, and methylglyoxal synthase. Notable, in the amino acid non-core was fatty acid metabolism (e.g. enoyl-CoA hydratase, 3- hydroxyacyl-CoA dehydrogenase, 3-ketoacyl-CoA thiolase, and butyryl-CoA dehydrogenase), and pyruvate metabolism I and II (e.g. succinate-semialdehyde dehydrogenase (NADP+), oxaloacetate decarboxylase alpha and beta chain, pyruvate dehydrogenase E1 component). The polypeptide non- core was e.g. enriched in serine glyoxylate cycle (e.g. methylmalonyl-CoA mutase) and folate- mediated one-carbon metabolism associated genes (e.g. methylenetetrahydrofolate dehydrogenase (NADP+), aminomethyltransferase, thymidylate synthase, and dihydrofolate reductase). The above-mentioned SEED subcategories covered the most abundant functional processes that were representative for carbohydrates and nucleic acids. However, the relatively high abundances of other subcategories were notable, especially in the protein treatments. In general, the protein cores were small accounting for ~1.5% of total reads with enrichments in DNA repair (~0.4% of total reads), *Phosphate metabolism* (~0.2%), and *RNA degradation* (~0.2%). However, non-cores were large in comparison accounting for ~20% of the total reads. The amino acid non-core was dominated by n- phenylalkanoic acid degradation (2.6%), Oxidative stress response (1.7%), and high abundances of 3- ketoacyl-CoA thiolase and Butyryl-CoA dehydrogenase (both ~0.8%) that are important in e.g. the degradation of amino acids such as valine, leucine, and isoleucine. Contrary, polypeptide non-cores were characterized by DNA-replication (0.9% of total reads), Purine de novo biosynthesis (0.8%) e.g. amidophosphoribosyltransferase (0.2%) involved in purine metabolism and alanine, aspartate and glutamate metabolism together with glucosamine--fructose-6-phosphate aminotransferase (~0.2%).
