# Supplementary figures and images for: Labile Dissolved Organic Matter Compound Characteristics Select for Divergence in Marine Bacterial Activity and Transcription
Source: Front Microbiol. 2020 Sep 25;11:588778. doi: 10.3389/fmicb.2020.588778 (PMC7546218; doi:10.3389/fmicb.2020.588778)

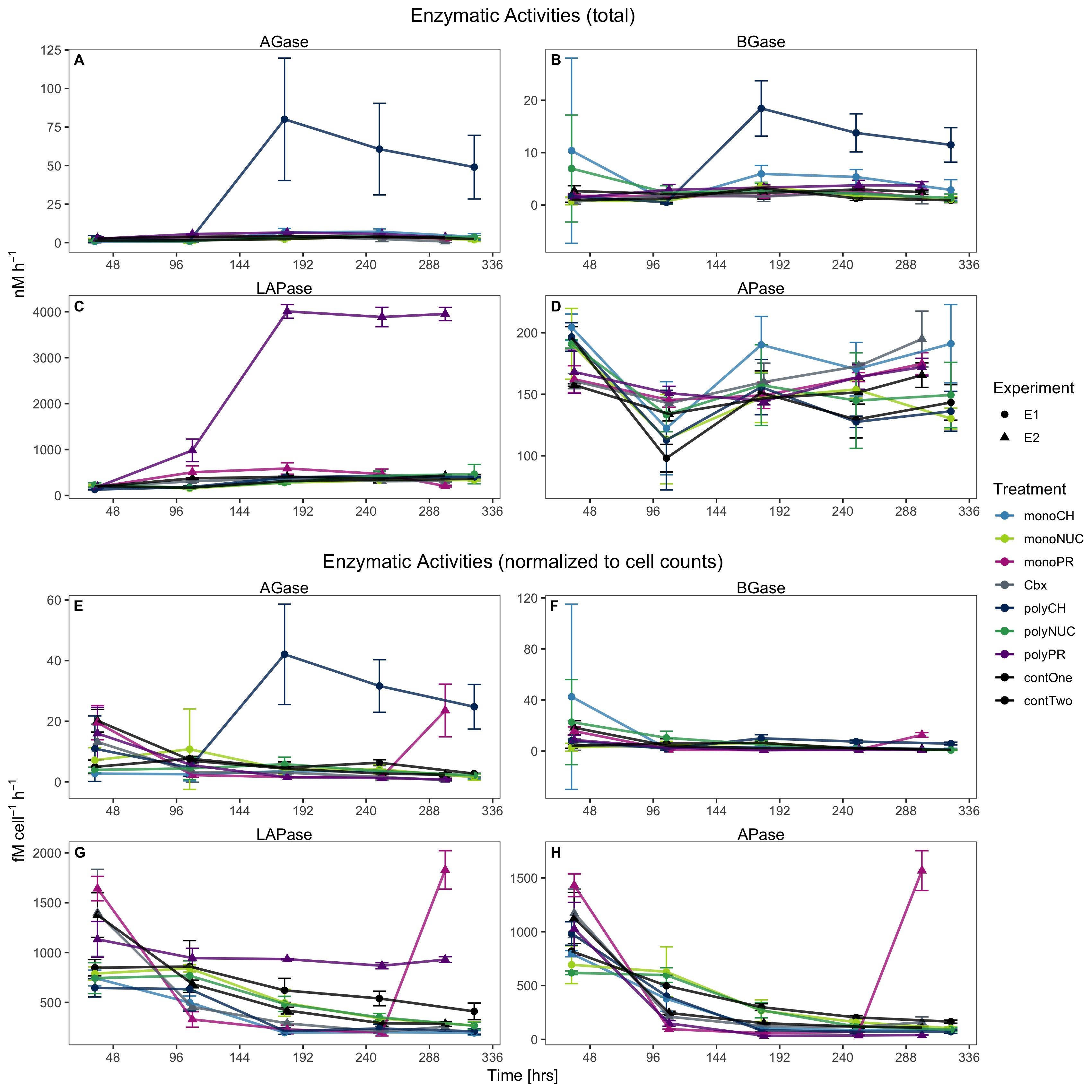

Supplement: Supplementary file 4 [file Image_2.jpeg]

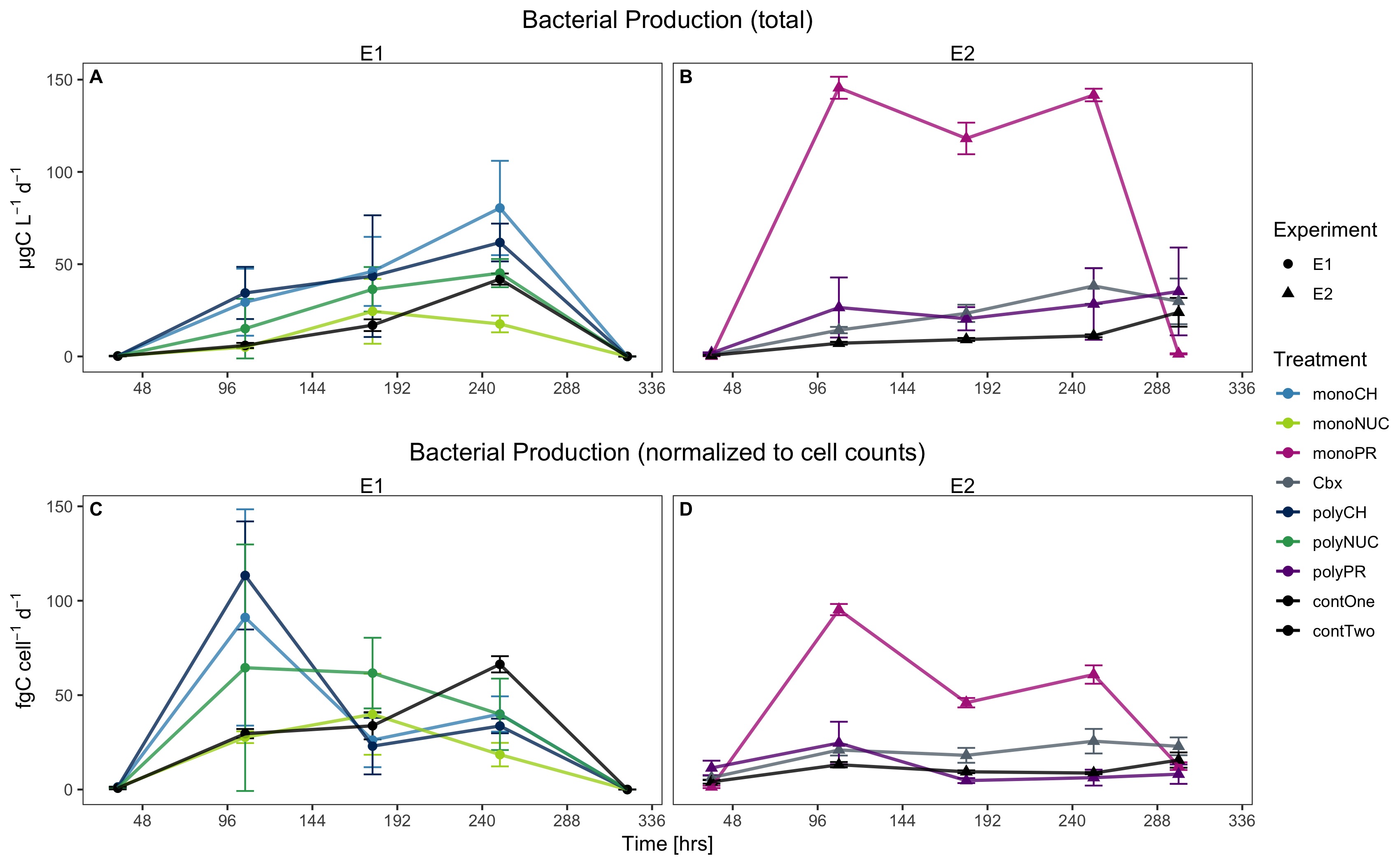

Supplement: Supplementary file 5 [file Image_3.jpeg]

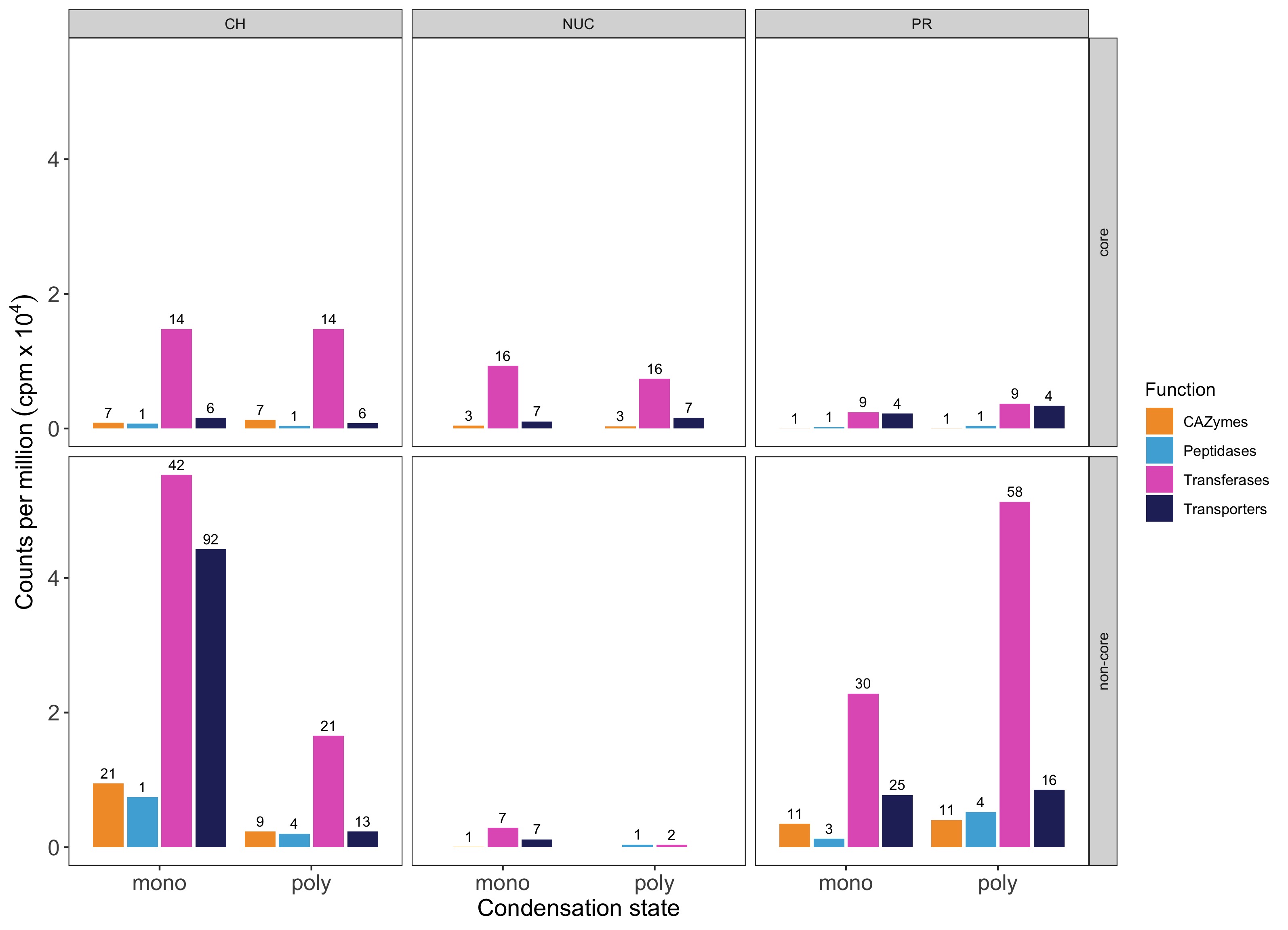

Supplement: Supplementary file 6 [file Image_4.jpeg]

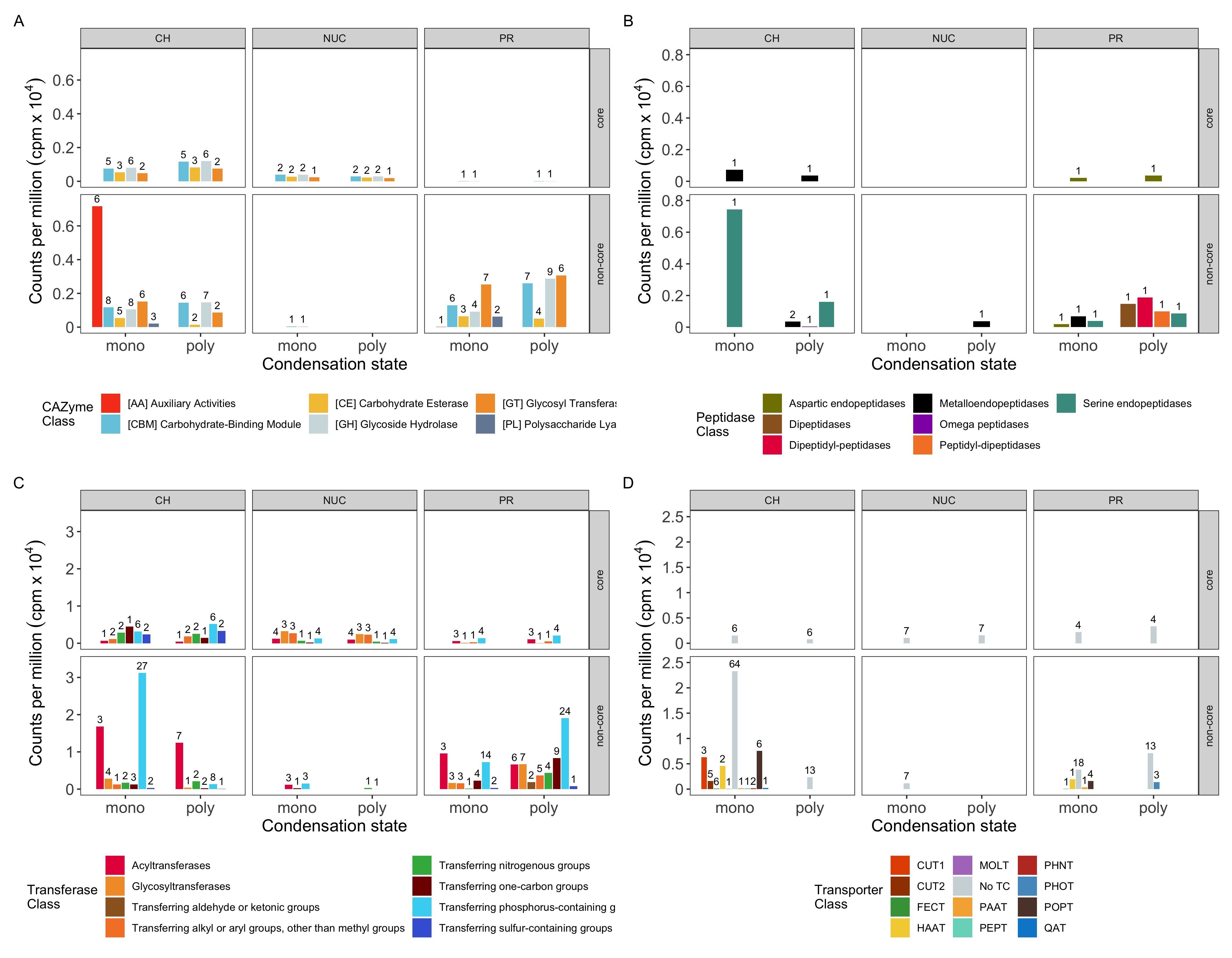

Supplement: Supplementary file 7 [file Image_5.jpeg]

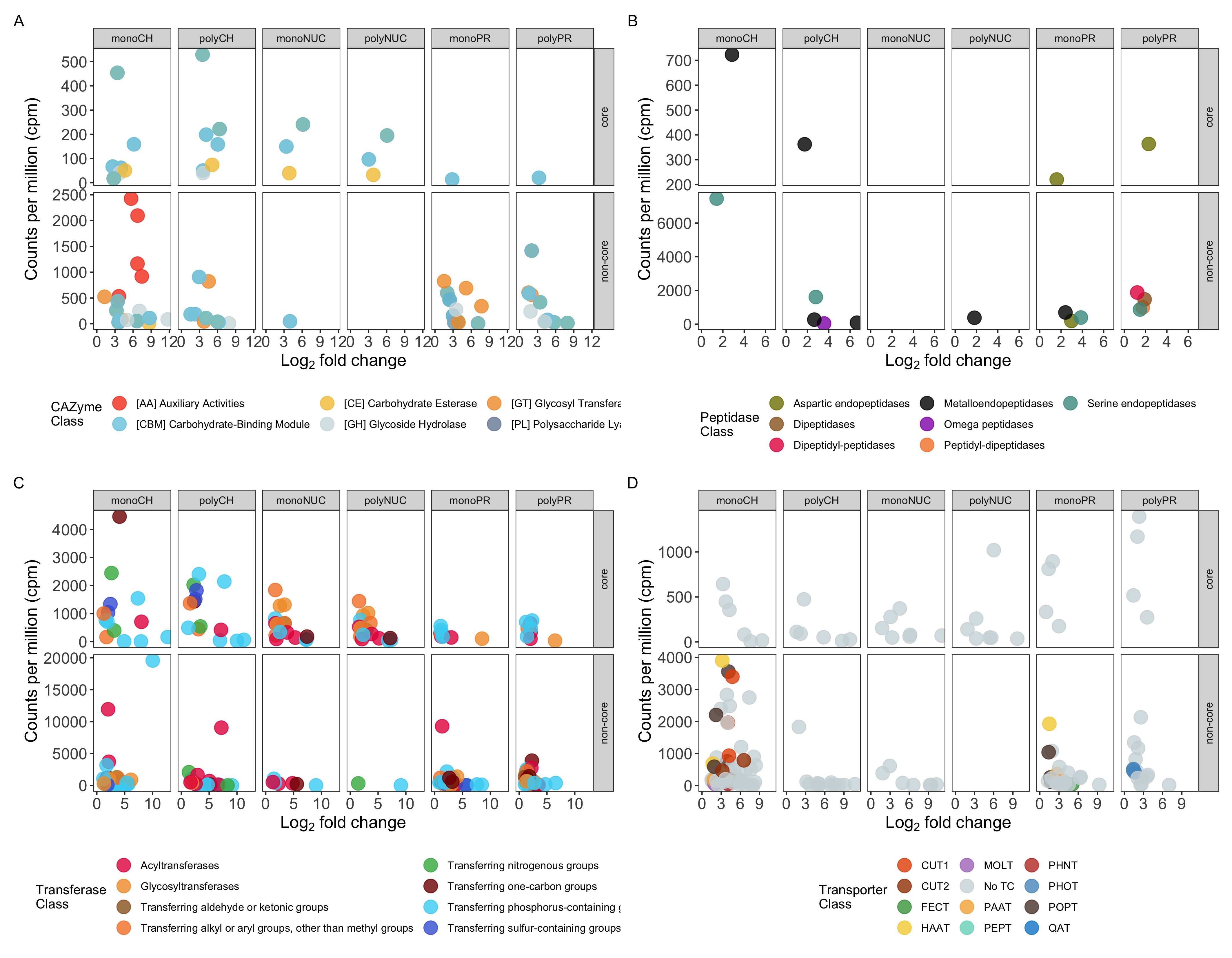

Supplement: Supplementary file 8 [file Image_6.jpeg]
